# MetaWRAP—a flexible pipeline for genome-resolved metagenomic data analysis

**DOI:** 10.1186/s40168-018-0541-1

**Published:** 2018-09-15

**Authors:** Gherman V. Uritskiy, Jocelyne DiRuggiero, James Taylor

**Affiliations:** Department of Biology, Johns Hopkins University, 3400 N Charles St., Baltimore, MD 21218 USA

**Keywords:** Metagenomics, WGS, Metagenome, Binning, Bin, Draft genome, Pipeline, Reassembly

## Abstract

**Background:**

The study of microbiomes using whole-metagenome shotgun sequencing enables the analysis of uncultivated microbial populations that may have important roles in their environments. Extracting individual draft genomes (bins) facilitates metagenomic analysis at the single genome level. Software and pipelines for such analysis have become diverse and sophisticated, resulting in a significant burden for biologists to access and use them. Furthermore, while bin extraction algorithms are rapidly improving, there is still a lack of tools for their evaluation and visualization.

**Results:**

To address these challenges, we present metaWRAP, a modular pipeline software for shotgun metagenomic data analysis. MetaWRAP deploys state-of-the-art software to handle metagenomic data processing starting from raw sequencing reads and ending in metagenomic bins and their analysis. MetaWRAP is flexible enough to give investigators control over the analysis, while still being easy-to-install and easy-to-use. It includes hybrid algorithms that leverage the strengths of a variety of software to extract and refine high-quality bins from metagenomic data through bin consolidation and reassembly. MetaWRAP’s hybrid bin extraction algorithm outperforms individual binning approaches and other bin consolidation programs in both synthetic and real data sets. Finally, metaWRAP comes with numerous modules for the analysis of metagenomic bins, including taxonomy assignment, abundance estimation, functional annotation, and visualization.

**Conclusions:**

MetaWRAP is an easy-to-use modular pipeline that automates the core tasks in metagenomic analysis, while contributing significant improvements to the extraction and interpretation of high-quality metagenomic bins. The bin refinement and reassembly modules of metaWRAP consistently outperform other binning approaches. Each module of metaWRAP is also a standalone component, making it a flexible and versatile tool for tackling metagenomic shotgun sequencing data. MetaWRAP is open-source software available at https://github.com/bxlab/metaWRAP.

**Electronic supplementary material:**

The online version of this article (10.1186/s40168-018-0541-1) contains supplementary material, which is available to authorized users.

## Background

The study of microbial communities through whole-metagenome (WMG) shotgun sequencing opens new avenues for the investigation of the metabolic potentials of microbiomes, in addition to their taxonomic composition [1–3]. This greatly improves the ability to interpret and predict functional interactions, antibiotic resistance, and population dynamics of microbiomes, with applications in human health, waste treatment, agriculture, and environmental stewardship [4–6]. WMG shotgun sequencing reads from hundreds to thousands of community members generate unique challenges for data analysis and interpretation [3, 7]. Software for WMG data analysis have grown in number and complexity, improving our ability to process, analyze, and interpret such data [8–12]. However, these tools are burdensome for biologists to work with. As the field of WMG expands, comprehensive and accessible software for unified analysis of metagenomic data is needed [7, 11].

Running a WMG analysis requires investigators to find the best currently available tools, install and configure them, address conflicting libraries and environment variables, and write scripts to convert outputs from one tool into the correct format to input into the next tool [13, 14]. These challenges present a major burden to anyone attempting metagenomic analysis, especially for investigators without computational experience, hindering progress of microbial genomics as a field [15]. Existing automated pipelines and cloud services lack modularity, do not give users control over the analysis, and often lack functions for genome-resolved metagenomics, the extraction of putative genomes (bins) through the binning of metagenomic assemblies [14, 16–19].

Genome-resolved metagenomics allows for reconstruction of the functional potential  of individual taxa and microbiome comparison at a finer scale. While a number of sophisticated tools such as CONCOCT, MaxBin, and metaBAT have been developed to address binning, it is still an actively improving field [9, 19–21]. Qualities of a metagenomic bin are (1) completion, the level of coverage of a population genome, and (2) contamination, the amount of sequence that does not belong to this population from another genome. These metrics can be estimated by counting universal single-copy genes within each bin [22, 23]. CheckM improves on this by checking for single-copy genes that a genome of the bin’s taxonomy is expected to have [24]. The percentage of expected single-copy genes that is found in a bin is interpreted as its completion, while the contamination is estimated from the percentage of single-copy genes that are found in duplicate.

Most metagenomic binning tools extract bins by clustering together scaffolds that have similar sequence properties, such as K-mer composition and codon usage, and similar read coverages across multiple samples [25, 26]. Because no single binning approach is superior in every case, bin consolidation tools attempt to combine the strengths and minimize the weaknesses of different approaches. DAS_Tool predicts single-copy genes in all the provided bin sets, aggregates bins from different binning predictions, and extracts a more complete consensus bin from each aggregate such that the resulting bin has the most single-copy genes while having a reasonably low number of duplicate genes [27]. This collapsing approach significantly improves the completion of the bins. Binning_refiner, on the other hand, splits the contigs into more bins such that no two contigs are in the same bin if they were in different bins in any of the original bin sets. This breaks the contigs into many more bins, reducing contamination [28]. Both of these approaches consolidate sets of bins from different methods and result in a superior bin set, but they have limitations—DAS_Tool increases completion at the expense of introducing contamination, while Binning_refiner prioritizes purity but loses completeness. Another way to improve draft genome quality that is relatively unexplored is bin reassembly—extracting reads that belong to a given bin and assembling them separately from the rest of the metagenome. With proper benchmarking, this approach could significantly improve the quality and downstream functional annotation of at least some bins in a microbial community.

Because the field of shotgun metagenomics is relatively new, there is a lack of software to inspect, analyze, and visualize metagenomic bins. While there are tools that can accurately predict the taxonomy of metagenomic scaffolds (such as Taxator-tk), there is no tool to classify entire metagenomic bins [29, 30]. Similarly, there are many ways to estimate the coverage of scaffolds based on read alignment depth but no way to find the coverages of entire bins across many samples [31, 32]. Finally, there is no tool to visualize draft genomes in context of whole metagenomic communities. The need for an easy-to-use integrated tool for WMG data analysis, as well as the lack of available tools for metagenomic bin analysis, motivated the construction of MetaWRAP.

## Implementation

### Main wrapper function

**Fig. 1:**
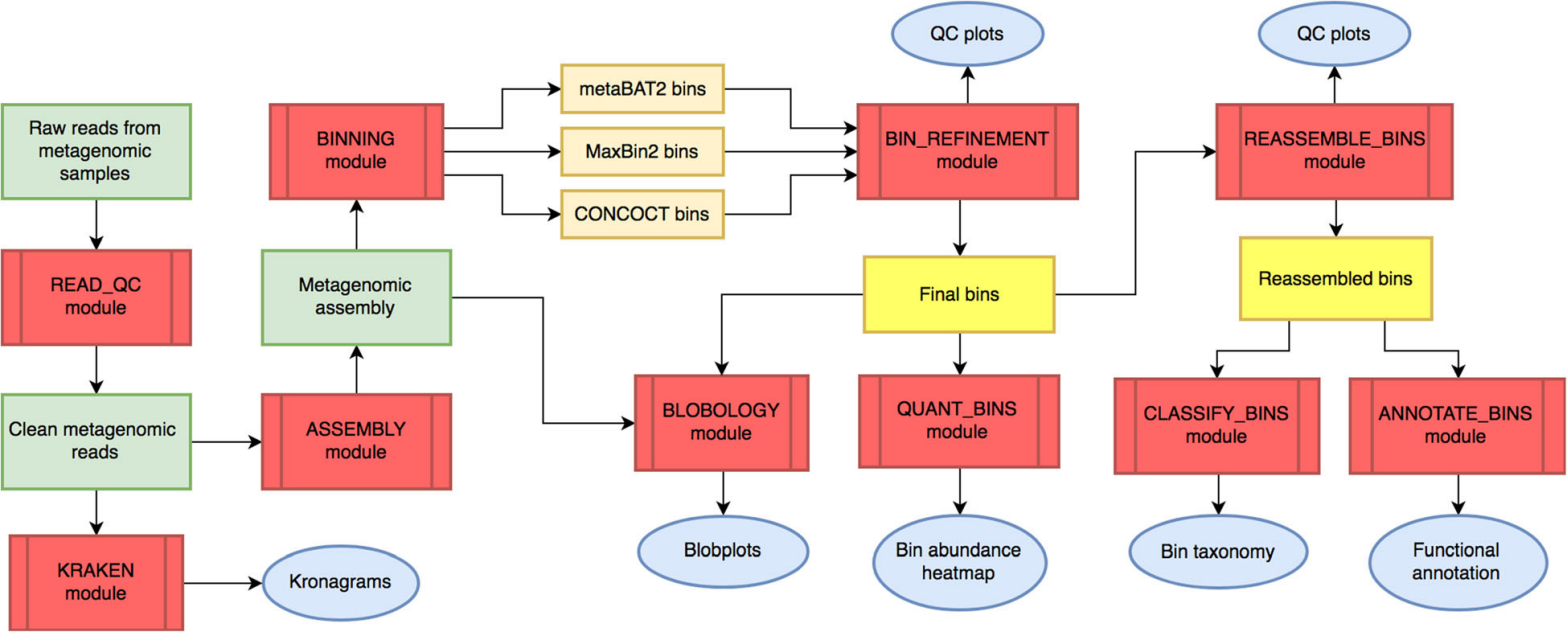
Overall workflow of metaWRAP. Modules (red), metagenomic data (green), intermediate (orange) and final bin sets (yellow), and data reports and figures (blue)

MetaWRAP is command line software for Unix-based systems that calls on a collection of modules, each being a standalone program addressing one aspect of WMG data processing or analysis (Fig. 1). Each module is a shell script pipeline that takes in a variety of input file parameters through command line flags. For detailed outlines of the algorithms behind each module, see supplementary material (Additional file 1). The modules call upon numerous installed software as well as custom Python 2.7 scripts (Additional file 2: Figure S1). MetaWRAP relies on the module folder (metawrap-modules), the script folder (metaWRAP-scripts), and a file containing paths to databases (config-metawrap) to be available in the PATH. MetaWRAP is hosted on github (https://github.com/bxlab/metaWRAP), distributed through Anaconda [33], and can be easily installed locally and on remote clusters. The metawrap-mg conda package (https://anaconda.org/ursky/metawrap-mg) includes metaWRAP and the necessary software for running any metaWRAP modules. The databases required by some modules need to be downloaded and unpackaged as described in the metaWRAP database download guide (https://github.com/bxlab/metaWRAP/blob/master/installation/database_installation.md) and their paths indicated in the config-metawrap file. MetaWRAP v0.7 was used in all benchmarking runs.

### Bin_refinement module

The metaWRAP-Bin_refinement module produces a superior bin set from multiple original binning predictions. First, hybrid bin sets are produced with Binning_refiner [28], which splits the contigs such that no two contigs are together if they were in different bins in any of the original sets. Then, the module goes over the different variants of each bin found in the original and hybridized bin sets and choses its best version based on completion and contamination metrics estimated with CheckM [24]. The decision of the “best bin” is based on the user-provided minimum completion and maximum contamination parameters. The contigs in the final bin set are then de-replicated, and a report of their completion, contamination, and other metrics is produced (Additional file 3: Figure S2). See supplementary methods (Additional file 1) for more details on the Bin_refinement module.

### Reassemble_bins module

The metaWRAP-Reassemble_bins module improves a set of bins by individually re-assembling each bin (Additional file 4: Figure S3). Reads are mapped to the bins with BWA v0.7.15 [32] strictly (no mismatches) and permissively (< 5 mismatches) and stored into their respective FastQ files. Importantly, read pairs will be pulled out even if only one read is aligned to the bin. Each read set is then reassembled with SPAdes [34], which produces more contiguous sequences compared to metagenomic assemblers such as MegaHit [35] and metaSPAdes [36] used in the Assembly modules. CheckM [24] is used to evaluate the completion and contamination of each of the three versions of each bin—the original bin, the “strict” re-assembled bin, and “permissive” re-assembled bin—and the best version of each bin is chosen for the final bin set based on the user-defined desired bin quality. See supplementary methods (Additional file 1) for more details on the Reassemble_bins module.

## Results and discussion

### MetaWRAP is a flexible, modular pipeline

The metaWRAP installation produces a bioinformatics environment with over 150 commonly used bioinformatics software and libraries (Additional file 2: Figure S1). MetaWRAP itself is a collection of modules, each of which uses a variety of pre-existing and newly developed software and databases to accomplish a specific step of metagenomic analysis. Unlike existing metagenomic wrappers and cloud services, metaWRAP retains modularity and grants the user control of the analysis pipeline. The user may follow the intuitive workflow starting from raw metagenomic shotgun sequencing reads all the way to high-quality draft genomes and their functional annotation or use only specific functions, as each module is also a standalone program (Fig. 1).

First, the metaWRAP-Read_qc module trims the raw sequence reads, removes human contamination, and produces quality reports for each of the sequenced samples. The reads from all given samples can then be assembled with the metaWRAP-Assembly module using MegaHit [35] or metaSPAdes [36], which also produces an assembly report. Both the reads from each sample and the assembly can be rapidly taxonomically profiled with the Kraken [29] module, producing interactive kronagrams [37] of community taxonomy. It should be noted that while Kraken is fast, post-classification standardization may be needed to obtain a more accurate community composition estimate [38]. The assembly is then binned with the metaWRAP-Binning module by three metagenomic binning software—MaxBin2, metaBAT2, and CONCOCT [19–21].

The other modules of metaWRAP focus on refining, analyzing, and visualizing metagenomic bins from either the Binning module or other sources. The metaWRAP-Bin_refinement module consolidates multiple binning predictions into a new, improved bin set, while also proving metrics of their completion and contamination. MetaWRAP-Reassemble_bins can then be used to reassemble the reads belonging to each bin, improving their N50, completion, and contamination. The resulting bins can be visualized by using the metaWRAP-Blobology module [39], which plots the contigs of the joint assembly on a blob plot, annotating them with their taxonomy and bin membership. The metaWRAP-Quant_bins module can be used to quickly estimate the abundance of each bin in each of the metagenomic samples. MetaWRAP-Classify_bins can be used to conservatively but accurately estimate their taxonomy. Finally, the bins can be functionally annotated with the metaWRAP-Annotate_bins module.

### Compute time of metaWRAP modules

The runtime of each metaWRAP’s modules was evaluated on a subset of the Human Intestinal Tract (MetaHIT) survey [40]. The same subset is used in the metaWRAP tutorial page https://github.com/bxlab/metaWRAP/blob/master/Usage_tutorial.md. The data contained three WMG samples, totaling 145.8 million 75 bp paired-end reads, or 21.9 Gbp of sequencing data. MetaWRAP was used to analyze this data set on a Linux server with 24 cores and 100 GB of RAM. All modules were run on default settings, and the total runtime of each module was recorded (Additional file 5: Module runtime). The entire pipeline was completed in 5 h and 36 min, with the majority of compute time dedicated to the Read_qc, Binning, Bin_refinement, and Reassemble_bins modules. With the exception of CONCOCT [19], the programs wrapped into metaWRAP can take advantage of multi-core systems and scale well with larger data sets. MetaWRAP itself also parallelizes processes when possible.

### MetaWRAP-Bin_refinement improved bin predictions in synthetic data

**Fig. 2:**
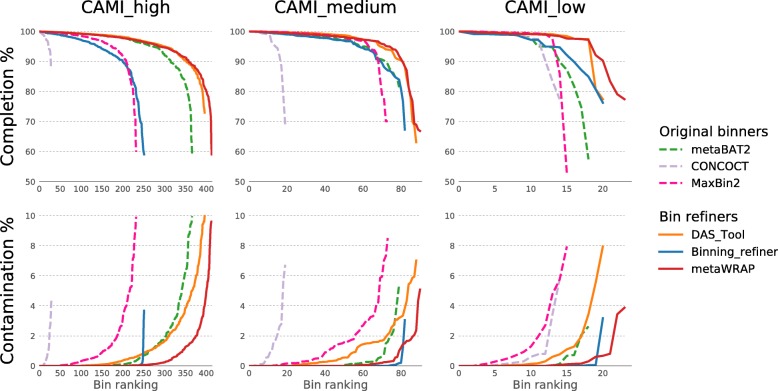
True completion and contamination of bins recovered from the CAMI’s high, medium, and low complexity synthetic data sets using original binning software (metaBAT2, MaxBin2, and CONCOCT) and software consolidating the original sets (DAS_Tool, Binning_refiner, and metaWRAP’s Bin_refinement module). Only bins with ≥ 50% completion and ≤ 10% contamination are shown

To test the efficacy of the metaWRAP-Bin_refinement module at consolidating and improving bin sets, we used synthetic metagenomic data sets of varying complexity from the Critical Assessment of Metagenomic Interpretation (CAMI) study [9]. The “gold standard” assemblies from the “high,” “medium,” and “low” diversity challenges were first binned with metaBAT2, Maxbin2, and CONCOCT [19–21] using the metaWRAP-Binning module, and the resulting three bin sets were then consolidated with DAS_Tool [27], Binning_refiner [28], and metaWRAP-Bin_refinement. The completion and contamination of the bins in the original and refined bin sets were evaluated with CheckM [24] (Additional file 6: Figure S4) and Amber [41] (Additional file 7: Figure S5). True recall and precision for each bin calculated with Amber were converted to completion and contamination percentages to be comparable to the CheckM results (Fig. 2). We found that metaBAT2 consistently outperformed MaxBin2 and CONCOCT, producing a total of 385 high-quality bins between all the challenges (completion greater than 90% and contamination less than 5%) and 271 near-perfect bins (completion greater than 95% and contamination less than 1%). MaxBin2 came in second with 275 high-quality bins and 164 near-perfect bins. CONCOCT performed rather poorly in all but the smallest CAMI challenge data sets, producing 58 high-quality bins and 40 near-perfect bins.

In the consolidated bin sets, DAS_Tool produced 426 high-quality bins and 263 near-perfect bins across all CAMI challenges, while Binning_refiner produced 289 and 210 bins, respectively. DAS_Tool consistently produced high-completion bins; however, these bins had relatively high contamination, which is a result of the aggregation approach that DAS_Tool takes. Binning_refiner on the other hand produced very pure bins with its splitting approach; however, it did so at the expense of significantly reduced completion. MetaWRAP-Bin_refinement produced bins that had both high completion and low contamination. In total, it produced 457 high-quality bins and 339 near-perfect bins (Fig. 2) due to both splitting and aggregation steps. These results confirmed that metaWRAP not only consistently improved bin sets through its consolidation approach, but it also outperformed other consolidation algorithms in data sets of varying complexity.

The CAMI challenge consisted of genomes of varying degrees of similarity and categorized the genomes into two broad categories depending on their average nucleotide identity (ANI) to other genomes in the mix. “Unique strains” are defined as genomes with < 95% ANI to any other genome and “common strains” as genomes with ≥ 95% ANI to another genome in the data set. [9] This gave us an opportunity to benchmark metaWRAP at recovering genomes from contig clusters of varying complexity. We found that metaWRAP outperformed all other binning methods in reconstituting both closely and distantly related genomes (Additional file 8: CAMI binning summary table). Interestingly, we found that Binning_refiner performed almost as well as metaWRAP in distantly related genomes but performed poorly in closely related genomes. On the other hand, DAS_Tool recovered almost as many closely related genomes as metaWRAP but performed relatively poorly in more discrete genomes.

The use of CheckM (Additional file 6: Figure S4) and Amber (Fig. 2) to evaluate the binning sets produced similar results, although overall CheckM slightly overestimated both completion and contamination of the produced bins. More importantly, the relative performance of the six binning approaches was the same when evaluating with CheckM or Amber. This validated the use of CheckM for benchmarking binning results in data sets where the true genomes remain unknown.

### Benchmarking metaWRAP on real metagenomes

MetaWRAP’s performance was also assessed with real WMG Illumina paired read sequencing data, using representative metagenomic data sets from water, gut, and soil microbiomes. The water data set was from a brackish water survey, which investigated the seasonal dynamics and biogeography of the surface bacterioplankton in the Baltic Sea [42]. This data set included 36 samples for a total of 196 Gbp of sequencing data. The gut data set came from the Metagenomic of the Human Intestinal Tract (MetaHIT) survey, which sequenced the gut microbiota from volunteers across Europe to explore the diversity and drivers in individual gut microbiome composition. [40]. The benchmarking data set consisted of 50 samples for a total of 144 Gbp of sequencing data. The soil data came from sequencing the highly diverse grassland soil microbial communities from Angelo Coastal Reserve, CA [27]. This data set consisted of six samples for a total of 481 Gbp of sequencing data.

**Fig. 3:**
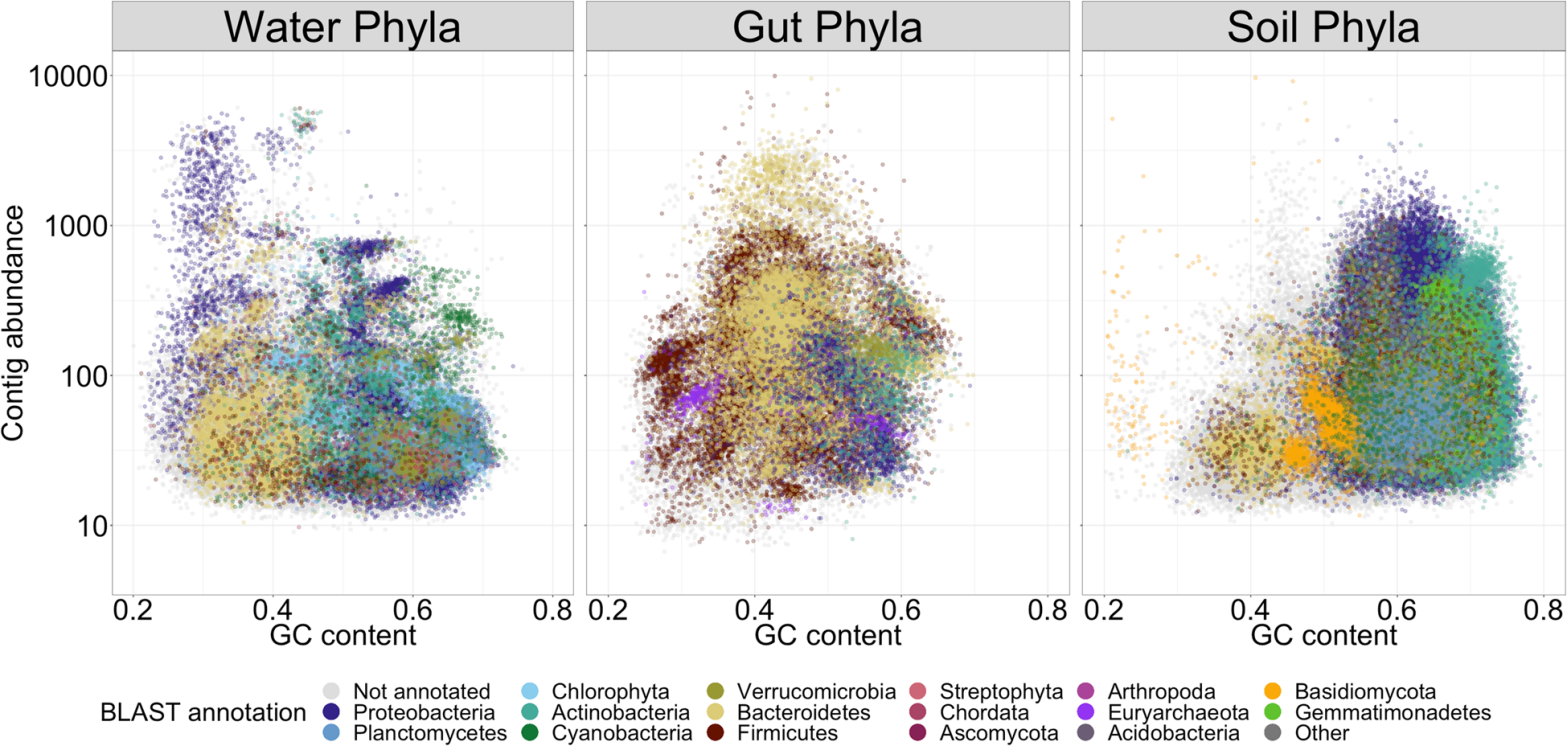
GC vs. abundance plots of contigs from the water, gut, and soil metagenomes, produced with the Blobology module. Abundance of contigs was calculated from standardized read coverage in each sample. Contigs were annotated with their phylum taxonomy, as determined by BLAST

Samples from each microbiome type were pre-processed through the metaWRAP-Read_qc module to trim reads and remove human contamination, and the Kraken and Blobology modules were used to evaluate the taxonomic profile of the communities. The water samples were dominated by *Proteobacteria*, the gut samples were dominated by *Bacteroidetes* and *Firmicutes*, and the soil samples comprised of a wide variety of *Proteobacteria* and *Actinobacteria* (Additional file 9). Notably, contigs from the soil microbiomes had much higher GC content compared to those of the gut and water. Also, soil contigs did not form as many defined clusters on the GC vs. abundance plot, suggesting that the communities were comprised of multiple closely related taxa (Fig. 3). Due to the high GC content and high taxonomic similarity of soil microbiota, this data set posed significant binning challenges compared to the water and gut microbiomes.

### Bin_refinement improved bin predictions in real data

**Fig. 4:**
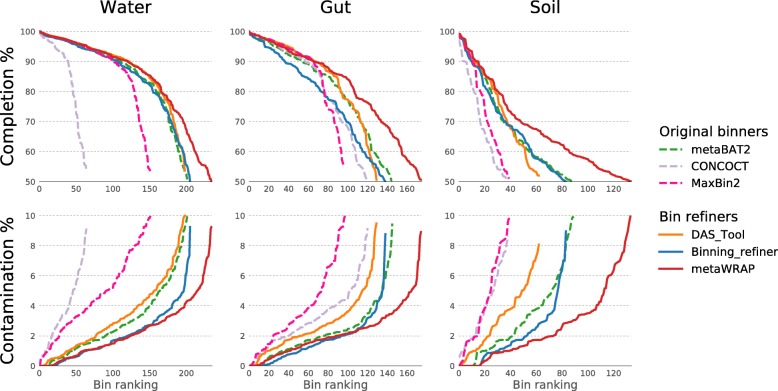
Completion and contamination of bins recovered from the water, gut, and soil metagenomes using original binning software (metaBAT2, MaxBin2, and CONCOCT) and software consolidating the original sets (DAS_Tool, Binning_refiner, and metaWRAP’s Bin_refinement module). Only bins with ≥ 50% completion and ≤ 10% contamination are shown (estimated by CheckM)

The quality-controlled reads from the representative metagenomic data sets were then co-assembled with the metaWRAP-Assembly module and the assemblies binned with metaBAT2 Maxbin2 and CONCOCT using the metaWRAP-Binning module. The resulting three bin sets for each microbiome type were consolidated with DAS_Tool, Binning_refiner, and metaWRAP-Bin_refinement, and the completion and contamination of the resulting bins were evaluated with CheckM (Fig. 4). Across the original binning software, metaBAT2 consistently produced the best sets of bins when compared to MaxBin2 and CONCOCT, with 202, 146, and 88 acceptable quality bins (comp ≥ 50%, cont ≤ 10%) in the water, gut, and soil samples, respectively. MaxBin2 had 151, 98, and 40 bins, and CONCOCT 65, 121, and 39 bins.

Despite incorporating all the binning methods, DAS_Tool was unable to improve the original bin sets, producing 198, 130, and 63 acceptable quality bins in the water, gut, and soil samples, respectively. DAS_Tool performed relatively well at higher bin completion ranges (≥ 80%), although at the expense of increased contamination. Binning_refiner performed similarly, with 206, 138, and 83 bins in the water, gut, and soil data sets, respectively. The bins from Binning_refiner were less complete but also had significantly lower contamination than bins in the original bin sets. MetaWRAP’s Bin_refinement module produced 235, 175, and 134 acceptable quality bins in the water, gut, and soil samples, respectively, significantly outperforming all other tested approaches. The module uses Binning_refiner in its pipeline to hybridize the input bin sets and then chooses the best version of each bin from the original and hybridized sets. Because the Bin_refinement module leverages the strength of Binning_refiner but still has a collapsing step similar to DAS_Tool, it is able to match DAS_Tool’s high-completion rankings, while retaining the low-contamination rankings of Binning_refiner. Overall, MetaWRAP consistently produced the highest quality bin sets in all the tested metagenomic data sets, which ranged greatly in diversity, taxonomic composition, and sequencing depths.

It is important to note that the use of metaWRAP’s Bin_refinement module to improve binning predictions is not limited to the bin sets produced from the metaWRAP-Binning module (metaBAT2, MaxBin2, and CONCOCT). Bin sets from any two or three binning software may be used as input for the module. Furthermore, because the algorithm leverages the differences between the input bin predictions, it is also possible to use bin sets produced from different parameters of the same software as input.

### Bin_refinement adjusts to the desired bin quality

To consolidate the original and hybridized bin sets, metaWRAP-Bin_refinement chooses the best version of each bin based on their completion and contamination values. However, this selection is subjective and depends on what the user believes to be the “best bin.” The minimum completion (-c) and maximum contamination (-x) options are key parameters that greatly alter the quality of the bins produced, as the module will dynamically adjust its algorithms to produce the maximum number of bins in this range.

To demonstrate the effects of changing the -c and -x parameters of metaWRAP’s Bin_refinement module, we ran the original bin sets from the water, gut, and soil data sets with varying minimum completion (but fixed maximum contamination) (Additional file 10: Figure S6) and varying maximum contamination (but fixed minimum completion) (Additional file 11: Figure S7) parameters. When compared to the original Bin_refinement run (-c 50 -x 10), the module produced a greater number of bins at any given threshold when it was given custom -c and -x parameters. The improvements were especially noticeable at higher completion and lower contamination ranges. For example, MetaWRAP-Bin_refinement -c 90 -x 10 recovered 19, 18, and 1 (water, gut, and soil, respectively) extra bins with a minimum completion of 90%, when compared to the baseline -c 50 -x 10 run. Similarly, MetaWRAP-Bin_refinement with -c 50 -x 1 parameters extracted 8, 21, and 4 (water, gut, and soil, respectively) more bins at a maximum contamination of 1%, when compared to the baseline run. Unlike arbitrary and sometime confusing thresholding parameters in many other software, the minimum completion and maximum contamination options offer the user an intuitive way to parameterize metaWRAP’s Bin_refinement module to their needs. This leads to significant increases in the number of quality bins they are able to extract from their data.

It is important to note that while refinement of binning predictions results in high-quality bins when evaluated with single-copy gene numbers, they do not represent the genomes of single individuals in a community or even individual strains. In this context, a bin is simply the optimized taxonomic clustering of contigs, which themselves are representative consensus resulting from the clustering of reads belonging to closely related taxa. In the context of phylogeny, bins may represent individual strains, species, or even higher-order averaged taxa, depending on the level of heterogeneity of the community in question. In the literature, bins are sometimes referred to as population genomes [43], underlying the complex nature of bins. As described in the context of the CAMI challenge, the analysis of a community with mostly “unique strains,” i.e., distantly related organisms, will result in bins potentially representing species or even strains, whereas the analysis of a community with mostly “common strains,” i.e., closely related organisms, will result in more hybrid bins. In reality, most communities are an assemblage of both closely and distantly related taxa resulting in a range of bin qualities.

Because of this, contamination resulting from strain heterogeneity is expected [44], and the desired bin quality can be tailored to the requirements of the downstream applications. For accurate taxonomic assignment of bins, a low contamination is important (1–5%) but a high completion may not be (20–50% may be sufficient). For accurate reconstruction of metabolic potential on the other hand, it is more important to reconstruct the genome with a higher completion (90–98%), even at the expense of introducing contamination (5–10%), as long as the user understands that the resulting bins represent the averaging of closely related taxa. The parameterization will also be constrained by the characteristics of the microbiome in question. Communities with relatively low diversity, low strain heterogeneity, and low GC content (such as gut microbiomes) will yield bins with lower contamination and higher completion than those extracted from a community with high diversity, heterogeneity, and average GC content (such as soil microbiomes).

### Reassemble_bins significantly improved bin quality

MetaWRAP’s Reassemble_bins module improves a given set of bins through individual reassembly with SPAdes [34]. The module only replaces the original bins if the reassembled ones are better in terms of completion and contamination. Like the Bin_refinement module, the Reassemble_bins module takes in minimum completion (-c) and maximum contamination (-x) parameters to allow the user to define what they consider a “good” bin. The bins produced from the water, gut, and soil data with metaWRAP-Bin_refinement module runs (-c 50 -x 10) were run through the metaWRAP-Reassemble_bins module (-c 50 -x 10), and the resulting bins were re-evaluated with CheckM [24].

**Fig. 5:**
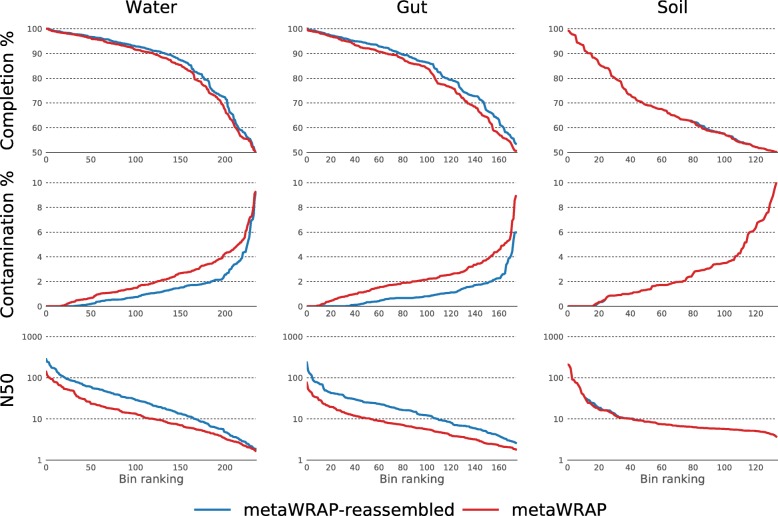
N50, completion, and contamination metrics of original bins extracted from the water, gut, and soil metagenomes with the metaWRAP’s Bin_refinement module and the same bins reassembled with metaWRAP’s Reassemble_bins module. Only bins with ≥ 50% completion and ≤ 10% contamination are shown (estimated with CheckM)

The Reassemble_bins module improved upon 78%, 98%, and 2% of the bins in the water, gut, and soil bin sets, respectively. The module significantly improved the water and gut bins’ overall metrics, increasing their N50 and completion scores. Even more strikingly, the reassembly process significantly reduced contamination in these bin sets (Fig. 5). The success of the bin reassembly algorithm relies heavily on accurate and specific recruitment of the correct reads to each bin. In very diverse and heterogeneous communities such as those found in soil, the read recruitment may not be specific enough. This confused the assembler during the re-assembly stage and resulted in an improvement for only a small fraction of the bins. However, draft genomes from the gut and water samples were still significantly improved with the Reassemble_bins module despite their complexity (Fig. 3). Just as with the binning process, it is important to note that the bins resulting from the reassembly do not represent the true genomes of individual organisms found in the community but are rather consensus backbones for reads coming from closely related organisms.

### MetaWRAP produced high-quality draft genomes

**Fig. 6:**
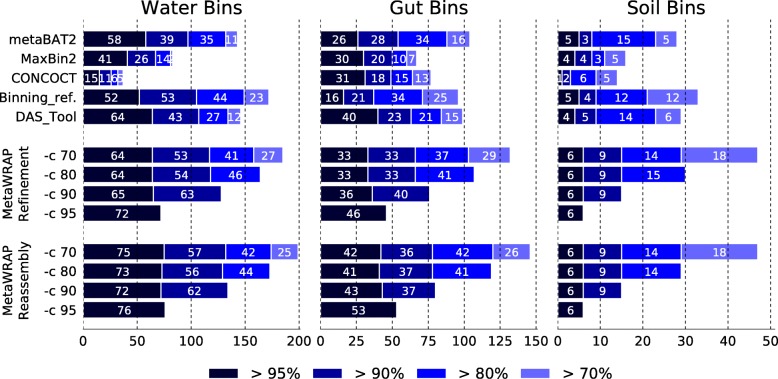
Number of high-purity bins (less than 5% contamination) extracted from the water, gut, and soil metagenomes with 70%, 80%, 90%, and 95% completion (estimated with CheckM) using original binning software (metaBAT2, MaxBin2, and CONCOCT) and bin-refining algorithms (Binning_refiner, DAS_Tool, metaWRAP-Bin_refinement, and metaWRAP-Reassemble_bins). MetaWRAP modules were run with varying -c (minimum completion) parameters. MetaWRAP’s Reassemble_bins module was run on the output of the Bin_refinement module

We investigated the performance of different binning approaches (both original binners and bin consolidation software) when extracting high-quality draft genomes, with a contamination less than 5% and completion greater than 70%, 80%, 90%, and 95% (Fig. 6). The default run of metaWRAP-Bin_refinement consistently produced the highest number of high-quality draft genomes in the water, gut, and soil data sets. These numbers further improved when re-running the module with appropriate minimum completion (-c) settings (i.e., running Bin_refinement -c 90 when benchmarking for bins with a minimum completion of 90%). This approach significantly outperformed every other tested binning and bin refinement method at every quality threshold.

The reassembly of the metaWRAP-derived bins with the Reassemble_bins module made a further improvement on the number of high-quality draft genomes extracted from the gut and water data sets. Even the default run of Reassemble_bins produced a significantly better bin set compared to non-reassembled bin sets produced by all tested software, including metaWRAP’s Bin_refinement. However, just like in the Bin_refinement runs, the results were further enhanced when Reassemble_bins was provided with an appropriate -c option.

When comparing to the original binning software (MaxBin2, metaBAT2, and CONCOCT) and bin consolidation tools (DAS_Tool and Binning_refiner), metaWRAP produced the largest number of high-quality draft genomes in all the tested WMG data sets. Additionally, it should also be considered that metaWRAP is capable of improving bin sets from any binning software. Therefore, when new metagenomic binning software are developed, their outputs can still be used with metaWRAP refinement and reassembly algorithms.

### MetaWRAP enables analysis and visualization of metagenomic bins

The rest of metaWRAP modules address examining and processing a set of bins in preparation for downstream analysis. The user may visualize the bins in the context of the entire community with the Blobology module, quantify their abundances across samples with the Quant_bins module, estimate their taxonomy with the Classify_bins module, and functionally annotate them with the Annotate_bins module.

The metaWRAP-Quant_bins module was used to estimate bin abundances across samples from their respective microbiome survey, and the results were shown in a clustered heatmap (Additional file 12: Figure S8). Clustered heatmaps may be used to infer bin co-abundance and to identify similarities and differences between samples. Because this approach considers the abundances of every extracted bin individually, it offers higher resolution information than when using higher taxonomic ranks.

**Fig. 7:**
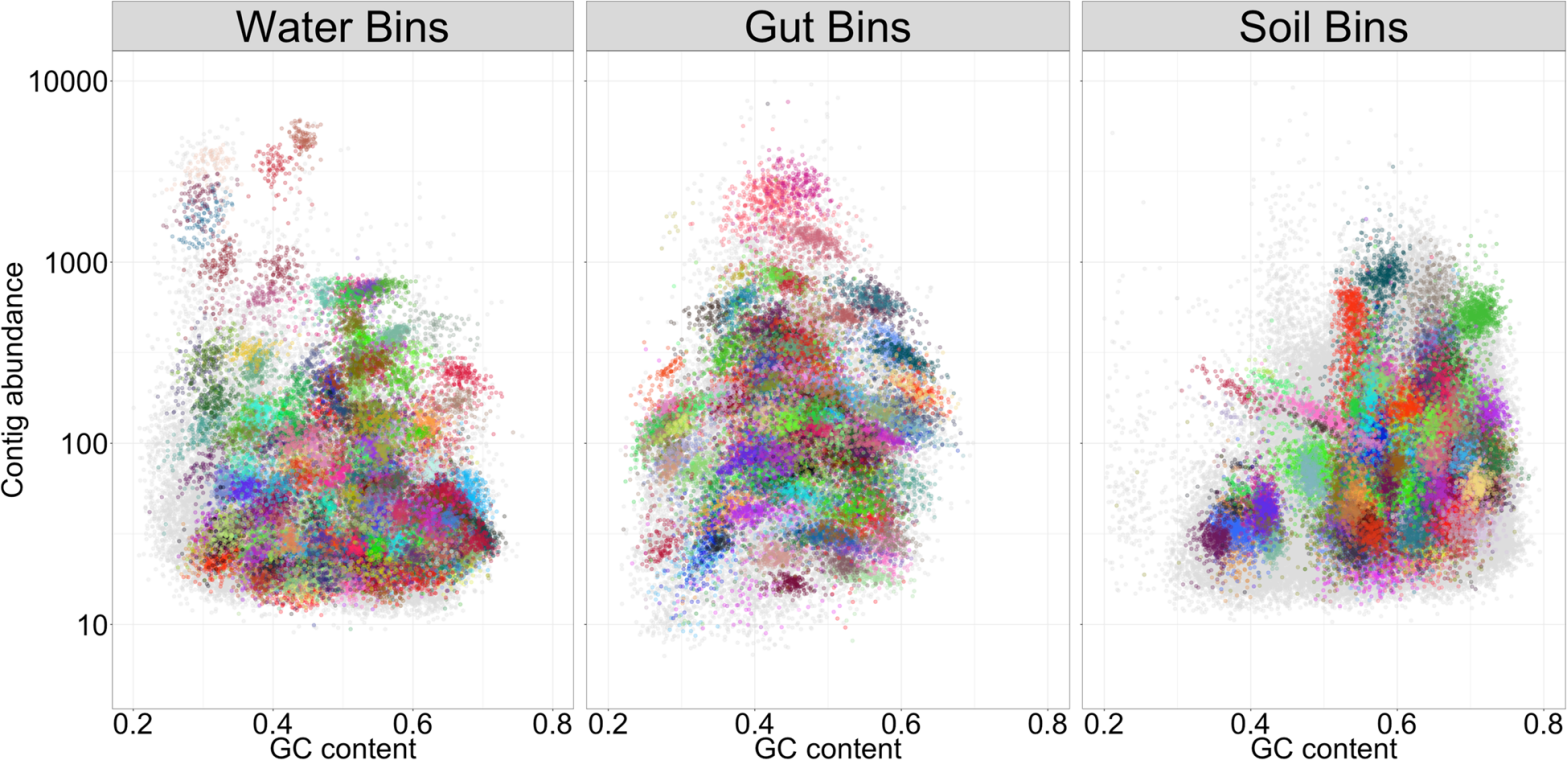
GC vs. abundance plots of contigs from the water, gut, and soil metagenomes, produced with the Blobology module. Abundance of contigs was calculated from standardized read coverage in each sample. The contigs were annotated with the bins that they belong to (bin colors are chosen at random), allowing for quick inspection of binning success. Bins were produced with metaWRAP’s Bin_Refinement module. Only bins with ≥ 70% completion and ≤ 10% contamination are shown (estimated with CheckM)

Bins were also visualized with the metaWRAP-Blobology module. The module produces GC vs. abundance plots of contigs, annotated with their taxonomy [45] (Fig. 3), bin membership (Fig. 7), or both (Additional file 13: Figure S9). These plots allow for inspection of the extracted bins in the context of the entire community that they belong to, as well as visualize the relative success of the binning process.

The final reassembled bins were taxonomy profiled with the metaWRAP-Classify_bins module (Additional file 14: Bin taxonomy) and functionally annotated with the Annotate_bins module. Together, this information may be used in downstream analysis to investigate complex questions about functional interactions and metabolic potential of individual community members.

## Conclusions

Genome-level analysis of WMG sequencing data is essential in understanding the composition and function of microbiomes. Until now, this rapidly growing field lacked a unifying platform to utilize the wealth of currently available software and make them easily accessible to researchers. MetaWRAP is a flexible pipeline that can handle common tasks in metagenomic data analysis starting from raw read quality control and ending in bin extraction and analysis. MetaWRAP is easy to install through Bioconda and simple to use, and its modularity gives the investigator flexibility in their analysis approach.

MetaWRAP contributed significant improvements to the recovery of draft genomes from shotgun metagenomic data through bin refinement and reassembly. The bin refinement module uses a novel hybrid approach to consolidate bin predictions from different binning software, producing a single stronger set. This approach significantly outperformed individual binning software, as well as other consolidation algorithms. The algorithm can adjust to accommodate specific draft genome quality targets, making it suitable for many research applications. MetaWRAP’s bin reassembly module further improved the draft genomes in both completeness and purity. Finally, metaWRAP contains multiple modules for analysis and evaluation of metagenomic bins—bin taxonomy assignment, abundance estimation, functional annotation, and visualization.

## Availability and requirements

Project name: metaWRAP

Project home page: https://github.com/bxlab/metaWRAP

Operating system: Linux64

Programming languages: Shell; Python 2.7

Other requirements: Conda; other packages automatically installed with metaWRAP: CONCOCT [19], MaxBin2 [20], metaBAT [21], CheckM [24], Binning_refiner [28], Kraken [29], Taxator-tk [30], BWA [32], SPAdes [34, 36], MegaHIT [35], KronaTools [37], Blobology [39], MegaBLAST [45], TrimGalore [46], BMTagger [47], FastQC [48], Bowtie2 [49], Salmon [50, 51], and PROKKA [52].

License: MIT

## Additional files

Additional file 1: Supplementary methods. Descriptions of analysis pipelines to process the benchmarking data, and detailed outlines of the algorithms in each metaWRAP module. (DOCX 170 kb)

Additional file 2: **Figure S1.** Detailed walkthrough of the data files, software, databases, and custom scripts that metaWRAP uses. The components of each metaWRAP module grouped and denoted with dotted lines. (PNG 2140 kb)

Additional file 3: **Figure S2.** Logical workflow of the Bin_refinement modules of metaWRAP. The module takes in three bin sets produced from the same assembly by different software or different parameters of the same software. Binning_refiner is used to create hybridized intermediates (four possible combinations), and the completion and contamination of the original and hybridized bins are estimated with CheckM. The best version of each bin is then found in the resulting seven bin sets. (PNG 123 kb)

Additional file 4: **Figure S3.** Logical workflow of the Reassemble_bins module, which extracts reads belonging to bins in a given bin set and individually reassembles them. This process is done for perfectly mapping reads (strict) and reads mapping with less than three mismatches (permissive). For each bin, CheckM is used to choose the best bin between the original and the two reassembled versions. (PNG 164 kb)

Additional file 5: Module runtime. The total real runtime of each module of metaWRAP when analyzing three samples from the metaHIT gut metagenomic survey. The modules were tested with default parameters on a Linux x64 server with 24 cores and 100 GB of RAM. (XLSX 23 kb)

Additional file 6: **Figure S4.** Completion and contamination (determined with CheckM) of bins recovered from the CAMI’s high, medium, and low complexity synthetic data sets using original binning software (metaBAT2, MaxBin2, CONCOCT) and software consolidating the original sets (DAS_Tool, Binning_refiner, metaWRAP). Only bins with ≥ 50% completion and ≤ 10% contamination are shown. (EPS 474 kb)

Additional file 7: **Figure S5.** True recall and precision (determined with AMBER) of bins recovered from the CAMI’s high, medium, and low complexity synthetic data sets using original binning software (metaBAT2, MaxBin2, CONCOCT) and software consolidating the original sets (DAS_Tool, Binning_refiner, metaWRAP). Only bins with ≥ 0.5% recall and ≥ 0.9% precision are shown. (EPS 474 kb)

Additional file 8: CAMI binning summary table. The number of bins recovered at different quality thresholds (determined with AMBER) from the CAMI challenge with original binning software (metaBAT2, MaxBin2, CONCOCT) and software consolidating the original sets (DAS_Tool, Binning_refiner, metaWRAP). MetaWRAP was run with default parameters. Performance is shown for “unique strain” (ANI < 95% to any other genome) and “common strain” (ANI > 95% to another genome) genomes. (XLSX 39 kb)

Additional file 9: Taxonomic distribution of reads from water, gut, and soil metagenomes, estimated with the metaWRAP-Kraken module. (HTML 972 kb)

Additional file 10: **Figure S6.** Completion of bins recovered from water, gut, and soil metagenomes with the metaWRAP-Bin_refinement module with a varying minimum completion parameter (-c) but constant maximum contamination parameter (-x 10). The numbers in the brackets indicate the number of extra bins gained at that threshold compared to the baseline run (-c 50 -x 10). Only bins with ≥ 50% completion and ≤ 10% contamination are shown. (EPS 394 kb)

Additional file 11: **Figure S7.** Contamination of bins recovered from water, gut, and soil metagenomes with the metaWRAP-Bin_refinement module with a varying maximum contamination parameter (-x) but constant minimum completion parameter (-c 50). The numbers in the brackets indicate the number of extra bins gained at that threshold compared to the baseline run (-c 50 -x 10). Only bins with ≥ 50% completion and ≤ 10% contamination are shown. (EPS 99 kb)

Additional file 12: **Figure S8.** Clustered heatmaps showing the log of bin abundance of bins extracted with metaWRAP-Bin_refinement (-c 50 -x 10) across samples in water, gut, and soil metagenomes, calculated and plotted with metaWRAP’s Quant_bins module. (PNG 974 kb)

Additional file 13: **Figure S9.** MetaWRAP-Blobology visualization of water, gut, and soil metagenomes, showing the GC and average coverage of each successfully binned contig (metaWRAP-Bin_refinement -c 70 -x 10) in the assemblies and annotated with the taxonomy at the phylum level and the bins that they belong to (bin colors are chosen at random). (PNG 2629 kb)

Additional file 14: Bin taxonomy. Distribution of the taxonomy among bacterial bins extracted from water, gut, and soil metagenomes using metaWRAP’s Bin_refinement module (-c 50 - x 10). Taxonomy estimated with metaWRAP’s Classify_bins module. (HTML 167 kb)

## Abbreviations

ANI: Average nucleotide identity
-c: Minimum completion parameter
comp: Completion
cont: Contamination
WMG: Whole metagenome
-x: Maximum contamination parameter
