# Supplementary material for: MetaWRAP—a flexible pipeline for genome-resolved metagenomic data analysis
Source: Microbiome. 2018 Sep 15;6:158. doi: 10.1186/s40168-018-0541-1 (PMC6138922; doi:10.1186/s40168-018-0541-1)
Supplement: Supplementary file 1 — Supplementary methods. Descriptions of analysis pipelines to process the benchmarking data, and detailed outlines of the algorithms in each metaWRAP module. (DOCX 170 kb) [file 40168_2018_541_MOESM1_ESM.docx]

BENCHMARKING METHODS

**CAMI binning benchmarking**

<https://github.com/bxlab/metawrap_paper/blob/master/running_metawrap_on_cami_data_commands.sh> contains the commands used for this analysis. Contigs from the “gold standard” assemblies from the “high”, “medium”, and “low” diversity CAMI challenges were binned with the metaWRAP Binning module (--metabat2 --maxbin2 --concoct parameters). The resulting bin sets were consolidated with DAS_Tool v1.1.0 (--search_engine blast parameter), Binning_refiner v1.2 (default settings), and metaWRAP Bin_refinement module (-c 50 -x 10 parameters). The completion and contamination of the bins in all six bin sets were first evaluated with CheckM v1.0.7 (default parameters), and bins with a completion less than 50% or a contamination greater than 10% were discarded. The true recall and precision of the bins within the six resulting bin sets was determined with Amber v0.6.2 and bin recall and precision were converted to completion and contamination percentages.

**Real data binning benchmarking**

<https://github.com/bxlab/metawrap_paper/blob/master/running_metawrap_on_real_data_commands.sh> contains the commands used for this analysis. The raw sequences from water, gut, and soil microbiomes were run through the metaWRAP-Read_qc module (default parameters) for quality trimming with TrimGalore, human contamination removal with BMTagger, and quality reports with FASTQC. MetaWRAP’s Kraken module (-s 10000000) was run on the quality-controlled reads with Kraken (using standard database) and KronaTools. The reads were co-assembled within each community type with metaWRAP-Assembly module (default parameters). Contigs shorter than 1000bp were discarded, with the exception of the soil assembly, for which the cutoff of 3000bp was chosen to reduce binning time. The contigs from the co-assemblies of each data type were binned with the metaWRAP-Binning module (--metabat2 --maxbin2 --concoct parameters). The resulting bin sets of each microbiome type were then passed to DAS_Tool v1.1.0 (--search_engine blast parameter), Binning_refiner v1.2 (default parameters), and metaWRAP-Bin_refinement module (-c 50 -x 10 parameters). The completion and contamination of all bins was estimated with CheckM v1.0.7 (default parameters).

**Bin_refinement optimization demonstration**

The metaWRAP-Bin_Refinement module was re-run to refine the bins produced by metaBAT2, MaxBin2, and CONCOCT bins from the water, gut, and soil microbiomes, but using different -c (minimum completion) and -x (maximum contamination) settings. First, the bin sets were refined with the module with a constant maximum contamination setting -x 10, but varying minimum completion settings -c 50, 60, 70, 80, 90, and 95. Then the same bin sets were refined with a constant minimum contamination setting -c 50, but varying maximum contamination setting of -x 10, 8, 6, 4, 2, and 1. The bin completion and contamination improvements were evaluated with CheckM v1.0.7 (default parameters).

**Reassembly benchmarking**

Bin sets produced from water, gut, and soil microbiomes by the metaWRAP-Bin_refinement module (-c 50 -x 10 parameters) were run through the metaWRAP-Reassemble_bins module (-c 50 -x 10 parameters). All reads coming from each respective microbiome was used for the reassembly. The bin completion and contamination improvements were evaluated with CheckM v1.0.7 (default parameters).

**Extracting high-quality draft genomes**

To test the performance of metaWRAP’s Bin_refinement module, it was run on the metaBAT2, Maxbin2, and CONCOCT bins with four different settings: -c 70 -x 5, -c 80 -x 5, -c 90 -x 5, and -c 95 -x 5. To test the Reassemble_bins module, it was run on the output of the Bin_refinement module runs. Bin_refinement was run with -c 60 -x 10, -c 70 -x 10, -c 80 -x 10, and -c 90 -x 10 settings, and then the resulting bins were reassembled with the Reassemble_bins module with -c 70 -x 5, -c 80 -x 5, -c 90 -x 5, and -c 95 -x 5 settings, respectively.

The bins were also refined with DAS_Tool v1.1.0 (--search_engine blast parameter), Binning_refiner v1.2 (default parameters). The completion and contamination of all bins was estimated with CheckM v1.0.7 (default parameters), and the number of bins with contamination less than 5% and completion greater than 70%, 80%, 90%, or 95% were counted.

**Draft genomes analysis**

Bins produced with metaWRAP-Bin_refinement (-c 70 -x 10 parameters) were visualized with the Blobology module (--bins flag used to provide bins), which uses a modified Blobology scripts, Bowtie2, and MegaBLAST to make Taxon-Annotated-GC-Coverage plots. Bin abundance in each sample was estimated and visualized with the Quant_bins module, which uses Salmon to quantify individual contigs and then estimate bin abundances. The reassembled bins from the metaWRAP-Reassemble_bins module (-c 50 -x 10 parameters) were run through the Classify_bins module (default parameters), which makes initial taxonomy predictions of individual scaffolds with Taxator-tk 1.3.3e and estimates the taxonomy of entire bins. Bins were functionally annotated with the metaWRAP-Annotate_bins module (default parameters), which uses PROKKA to annotate each bin.

METAWRAP MODULE DESCRIPTIONS

**Note:** metaWRAP’s modules take in maximum available thread count and memory parameters, and will pass this information to the wrapped programs when possible.

**MetaWRAP-Read_qc**

The Read_qc module is meant to pre-process raw Illumina sequencing reads in preparation for assembly and alignment. The raw reads are trimmed with Trim-galore v0.4.3 (--no_report_file and --paired settings), and then the human-derived reads (contamination) are removed with bmtagger v3.101 (default settings). Read pairs with a single suspected human read are also removed. FastQC (default settings) is then used to generate quality reports of the raw and final read sets in order to assess read quality improvement. The user has control over which of the above features he wishes to use.

**MetaWRAP-Assembly**

The Assembly module allows the user to assemble a set of metagenomic reads with either metaSPAdes v3.11.1 or MegaHit v1.1.2 (both at default settings). While metaSPAdes results in a superior assembly in most samples, MegaHit scales well with large datasets, and is therefore set as the default in the modules. The assemblies are then formatted to include the scaffold length and kmer depth, sorted by length, and contigs shorter than 1000bp are removed. An assembly report is then generated with QUAST v4.5 (default settings).

**MetaWRAP-Kraken**

The Kraken module takes in any number of FastQ or FastA files, classifies the contained sequences with KRAKEN v0.10.6 (default settings), and reports the taxonomy distribution in an interactive html kronagram using KronaTools v2.7 (default settings). If a passed FastA file is an assembly file from the Assembly module, the taxonomy of each contig is weighted based on its length and coverage [weight=coverage*length], which are encoded in the scaffold naming.

**MetaWRAP-Binning**

The Binning module is meant to be a convenient wrapper around three metagenomic binning software: metaBAT v2.12.1 (-m 1500 and --unbinned parameters), Maxbin v2.2.4 (-markerset 40 option), and CONCOCT v0.4.0 (default settings). First the metagenomic assembly is indexed and paired end reads from any number of samples are aligned to it with bwa v0.7.15 (defaults settings). The alignments are sorted and compressed with Samtools v1.6 (default settings), and library insert size statistics are also gathered at the same time (insert size average and standard deviation). MetaBAT2’s jgi_summarize_bam_contig_depths function is used to generate contig abundance table, and it is then converted into the correct format for each of the three binning software. The assembly is binned with software(s) of the user’s choice, and the resulting bins are optionally evaluated with CheckM v1.0.7 (default settings).

**MetaWRAP-Bin_refinement**

The Bin_refinement module utilizes a hybrid approach to take in two or three bin sets that were obtained with different binning approaches and produces a consolidated, improved bin set. First, binning_refiner v1.2 (default settings) is used to hybridize the bin sets in every possible combination. If there are three original bin sets A, B, and C, they will be hybridized to produce bin sets AB, BC, AC, and ABC. CheckM v1.0.7 (default settings) is then run to evaluate the completion and contamination of the bins in each of the 7 bin sets (3 originals, 4 hybridized). The bins sets are then iteratively compared to each other, and each pair is consolidated into an improved bin set. To do this, the same bin is identified within the two bin sets based on a minimum of 80% overlap in genome length, and the better bin is selected based on the scoring function S=Completion-5*Contamination. Only bins that meet the minimum completion (-c) and maximum contamination (-x) criteria are considered. After all bin sets are incorporated into a consolidated set, duplicate contigs are removed. By default, duplicate kept only in the superior bin (based on scoring function). CheckM is then re-run on the final bin set (default parameters) and a final report file is generated with a custom script (.stats files). Completion and contamination rank plots are also made to compare the quality of the original bins and the Bin_refinement module output.

**MetaWRAP-Reassemble_bins**

The Reassemble_bins module aims to improve a set of bins by extracting reads that belong to each bin and re-assembling them. First, entire original metagenomic assembly is indexed and FastQ reads are aligned back to it with bwa v0.7.15 (default parameters). Reads pairs mapping back to contigs belonging to the provided bins are stored in separate FastQ files, even if only one read mate aligned. Two sets of reads are stored for each bin – reads mapping perfectly (strict), and reads mapping with <3 mismatches (permissive). Each set of reads is then reassembled with SPAdes v3.11 (--careful setting), and short contigs (<1000bp) are removed. CheckM v1.0.7 (default settings) is used to evaluate the completion and contamination of each of the three versions of each bin – the original bin, the “strict” re-assembled bin, and “permissive” reassembled bin. The best version is chosen based on a scoring function S=Completion-5*Contamination. The final bins set it then re-evaluated with CheckM, and summary statistics are generated. Additionally, a N50, completion and contamination rank plots is generated to evaluate the improvements in the bin sets following reassembly.

**MetaWRAP-Quant_bins**

The Quant_bins module rapidly estimates the abundance of bins across a number of samples. Salmon v0.9.1 (--libType IU option) is used to index the entire metagenomic assembly and align reads from each sample back to the assembly. Coverage tables are generated estimating the abundance of each contig in each sample. The average abundance of each bin in each sample is calculated by taking the length-weighted average of the bins’s contig abundances. A final bin abundance table is made, and a clustered heatmap is generated with Seaborn v0.8.1 to visualize bin abundance variation across samples. The bin abundances are standardized to 100 million reads in each sample library before plotting.

**MetaWRAP-Blobology**

The Blobology module uses a modified version of the original Blobology software to create blobplots (a GC vs abundance plot of all the contigs) of a metagenomic assembly, and annotates it with phylogenetic information or bin information. The taxonomy of each contig is estimated with blastn v2.7.1 (-task megablast -evalue 1e-5 -max_target_seqs 1 -outfmt '6 qseqid sseqid staxids' parameters) with NCBI_nt as the database. The assembly is then indexed and the reads from any number of samples are aligned against it with bowtie2 v2.3.0 (--very-fast-local -k 1 -t --reorder --mm parameters). Blobology’s gc_cov_annotate.pl function is used to generate a blobplot file with the GC, coverage (in all samples), and taxonomy of each contig. If the user provided a set of bins to annotate, the contigs are also annotated with the bins they belong to. Finally, Blobology’s makeblobplot.R function is used to make the blobplots of the contigs across all the provided samples, with taxonomic and bin membership annotations.

**MetaWRAP-Classify_bins**

The Classify_bins module is a conservative way to assign taxonomy to a set of metagenomic bins. First, the contigs in all bins are combined into one file, and blastn v2.7.1 (-task megablast -outfmt '6 qseqid qstart qend qlen sseqid staxids sstart send bitscore evalue nident length' parameters) is used to align the contigs to the NCBI_nt database. The alignment results are then used by taxator-kt v1.3.3e (-a megan-lca -t 0.3 -e 0.01 parameters) to estimate the taxonomy of each contig. The most likely taxonomy of each bin is then estimated from individual contig predictions. Taxonomy of each contig are added to a phylogenetic tree, adding weight to each branch based on the length of that contig. The tree is then traversed from the root down the heaviest branches until the next likely branch is <50% of the current branch weight. Once no further taxonomic rank can be estimated, the final taxonomy of that bin is reported.

**MetaWRAP-Annotate_bins**

The Annotate_bins module takes in a set of bins and quickly functionally annotates them with PROKKA v1.12 (--quiet option). The annotation process is parallelized for any number of bins and threads. For each bin, the module returns the annotation file in GFF format, and two FastA files with untranslated and translated genes.
