# Supplementary figures and images for: MetaWRAP—a flexible pipeline for genome-resolved metagenomic data analysis
Source: Microbiome. 2018 Sep 15;6:158. doi: 10.1186/s40168-018-0541-1 (PMC6138922; doi:10.1186/s40168-018-0541-1)

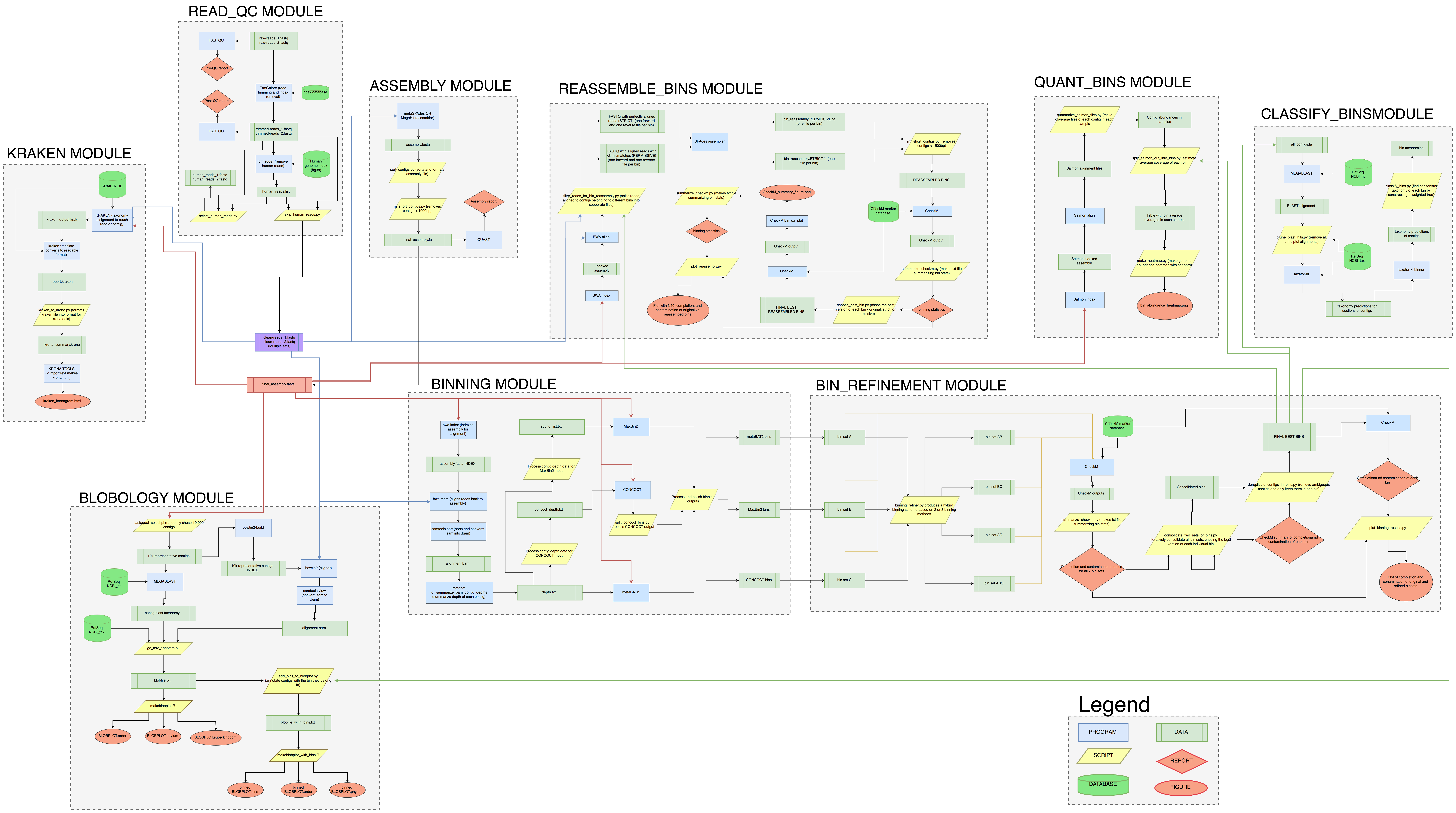

Supplement: Supplementary file 2 — Figure S1. Detailed walkthrough of the data files, software, databases, and custom scripts that metaWRAP uses. The components of each metaWRAP module grouped and denoted with dotted lines. (PNG 2140 kb) [file 40168_2018_541_MOESM2_ESM.png]

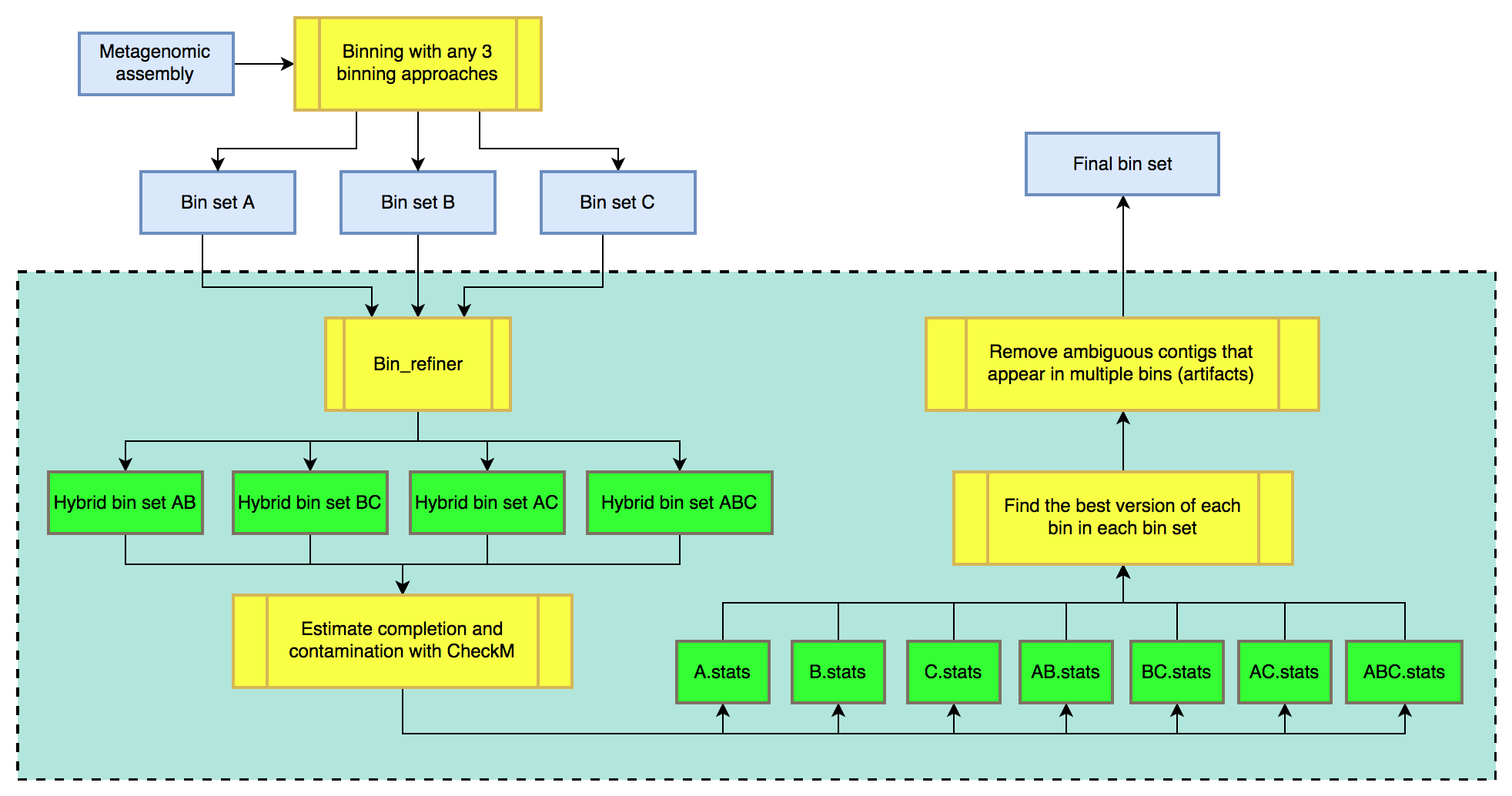

Supplement: Supplementary file 3 — Figure S2. Logical workflow of the Bin_refinement modules of metaWRAP. The module takes in three bin sets produced from the same assembly by different software or different parameters of the same software. Binning_refiner is used to create hybridized intermediates (four possible combinations), and the completion and contamination of the original and hybridized bins are estimated with CheckM. The best version of each bin is then found in the resulting seven bin sets. (PNG 123 kb) [file 40168_2018_541_MOESM3_ESM.png]

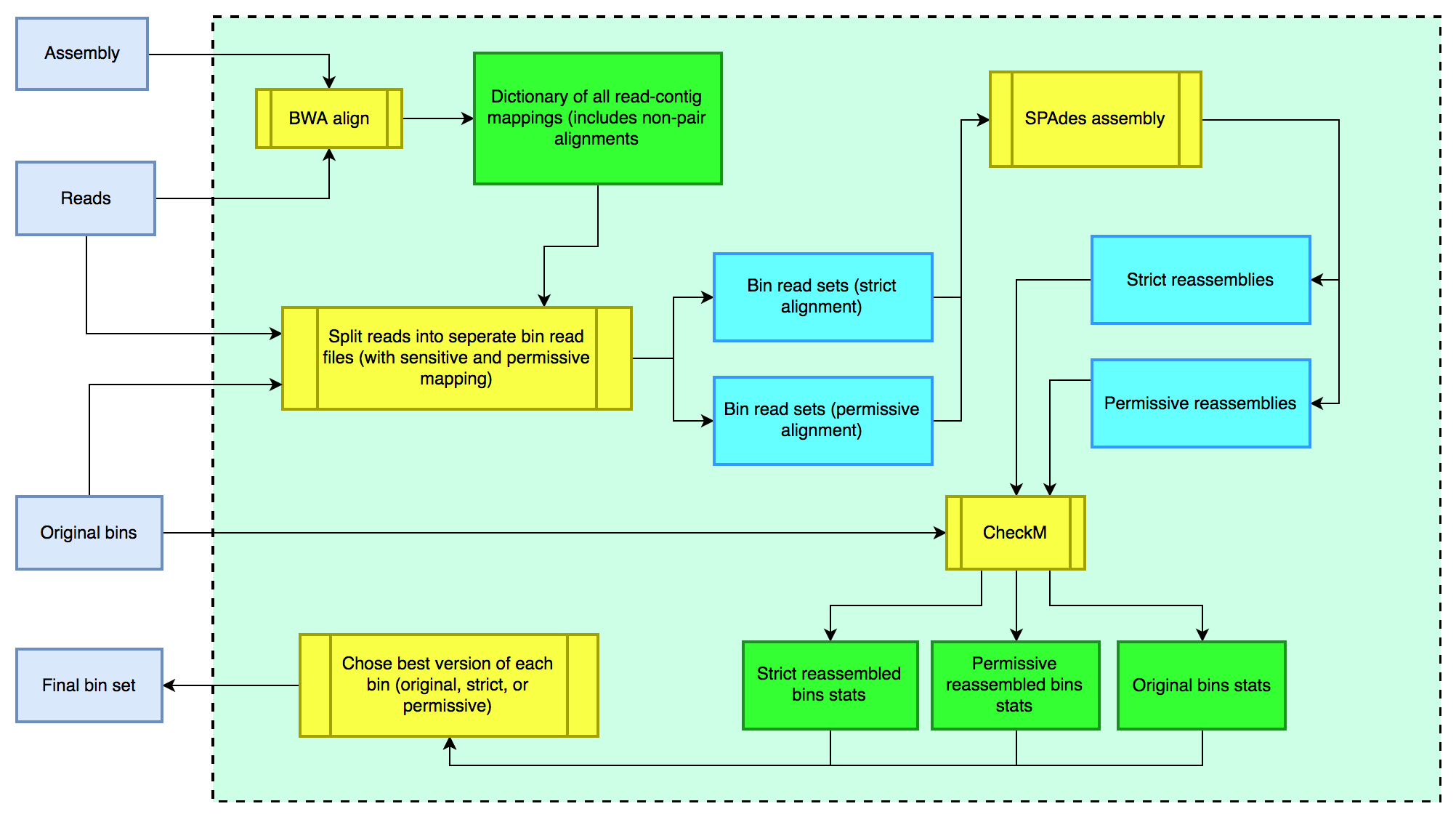

Supplement: Supplementary file 4 — Figure S3. Logical workflow of the Reassemble_bins module, which extracts reads belonging to bins in a given bin set and individually reassembles them. This process is done for perfectly mapping reads (strict) and reads mapping with less than three mismatches (permissive). For each bin, CheckM is used to choose the best bin between the original and the two reassembled versions. (PNG 164 kb) [file 40168_2018_541_MOESM4_ESM.png]

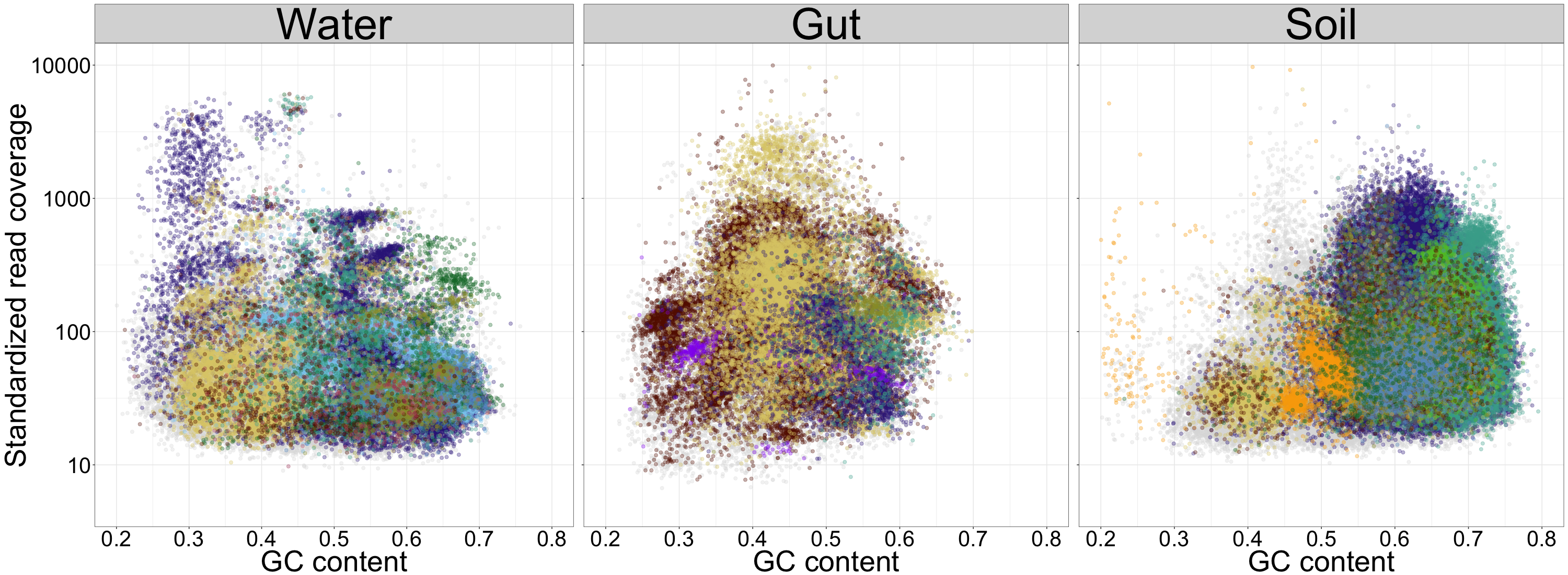

Supplement: Supplementary file 13 — Figure S9. MetaWRAP-Blobology visualization of water, gut, and soil metagenomes, showing the GC and average coverage of each successfully binned contig (metaWRAP-Bin_refinement -c 70 -x 10) in the assemblies and annotated with the taxonomy at the phylum level and the bins that they belong to (bin colors are chosen at random). (PNG 2629 kb) [file 40168_2018_541_MOESM13_ESM.png]
