# Supplementary material for: MetaWRAP—a flexible pipeline for genome-resolved metagenomic data analysis
Source: Microbiome. 2018 Sep 15;6:158. doi: 10.1186/s40168-018-0541-1 (PMC6138922; doi:10.1186/s40168-018-0541-1)
Supplement: Supplementary file 9 — Taxonomic distribution of reads from water, gut, and soil metagenomes, estimated with the metaWRAP-Kraken module. (HTML 972 kb) [file 40168_2018_541_MOESM9_ESM.html]

Javascript must be enabled to view this page.

members
magnitude
magnitudeUnassigned

Gut
Soil
Water

12313341249207377758

12313341249207377758
61754010565

101786
124324240257534

28

27

27

27

27

1

1

1

1

1

1

1

1

24137730606

3

3

1

2

24137730599

24137730599
132

24136430597

1

1

1

3

3

1

1

1

1

2691242

825

2

1

1

1

1

823

24

3

1

1

1

1

21

1

1

618

618

2683217
26

1

1

1

1

24

21

1
21

1

1

3

3

3

1

1

1

97

87

1

111

111

11

1

131

11

11

1

1

12

12

12

2

1

1

1

1

1

1

55162

45162

1

1

1

1

1

13

13

24762

1

1

1

14762

14762

1

1

1

1

1

32

32

21

11

31152

19

11

1

1

1

1

8

8

8

1

1

1

1

1

1

1

1143

1143

11

11

1

1

3

2
3

1

1

224236

512
2138

116
73

1

1

21

11

32

21

11

218

116

12

11

1

1

23

1

1

1

1

13

13

1

1

1

1

33

33

1

31

1

118386926463
601584

1170

5

5

5

124

124

12

12

3

2

1

441

1
439

210

210

228

2

951057
1

5319

5319

1
411038

29974

1164

51323419
5125

5741799
5149

227948

4100

1

100

100

3

959

33

3

2

138

216

3

3

3376
12

84

95

3185

211410

9

1660

15341

7126

174
2

11

2

33

126

127

114

2

11

521

521

4

3

1

1
335576

23

23

2

1

1

317143

2

2

3724

360

313

444

3302
40

60

11

194

297

297

15105

311

160

423

210

1

1

1

3

2134

2134

511360

52

52

33937
257

4182

9232

14346

4120

18371

18371

1

1

1

889

14

14

14

53

13

13

4

4

4

367

22

22

164

164

1

1

2

2

2

1

1

1

1

1

1

1

1

3
146675

11348

61

2

41

12

12

745

745

33500
3

13

13

13

7
19135

10

310

326

326

27

27

35

832

218

28

28

214

214

214

127
7105

153

113

33

33

19

359

359

65

58

7

39
1

29

9

9

1

1

47

47

3

44

2

2

124

124

124

539315
12

211

211

428161

326142

1219

1219

18141

18141

18141

13724

4
13724

10442

10442

246

246

1232

152461
1

272

272

132332

112309

223

56

56

56

1

1

1

26251

248

2

48

48

6203

3

3

284

247

247

37

37

273

14

2

2

137

11631563964
58

117712
108567496

12

12

93

9

2

3

1

1

1

1

3

1

1

1

1

346
86

53

62

46

29

17

53

703
60734474

110

16

1

1

2

4

1

1

2

1

1

1

6

1

1

2

1

2

11

1

148

1

1

1

71

1

73

1

1

1

105

1

4

1

1

1

3

31

3

4

1

11

1

1

4

4

1

6

1

1

30

1

2

2

1

1

1

1

305

1

1

1

1

2

2

4

2

35

224

1

1

212

1

1

1

3

1

1

4

4

1

1

3
2

1

1

1

2

1

1

71
6

1

1

5

4

1

5

5

1

1

315498
2

28223

5

11

5

3

53

1

66

4

1

1

19

49

1

1

17

4

1

1

2

2

1

1

2

2

6262

3207

3206

14

2200

2

1

1

1

1

1

1

254

1

2

251

2

1

1

1

1

1

15

1

13

1

1

1

1

87

25

1

1

60

1281
75742825

66

3

6

3

841434

3
841432

1120
731427

479

1

2

2

41

51

1

28

22

7

97

2

45

60

77

1

358

1260

28

50

11

1

1

1

2

2

2

1

1

2

2

11

11

1

1

1

10
14161

13

13

114

2

12

1

12134

11

1134

8

1126

221

11

21

5

5

7157
51551145

1

25

1100

1100

16

28

344

12

46

46

17

1

52

3

30

310

5

4

71

1

1

1

26

121

1

1

112

1

43

3

1

30

52554

1

1

8

71

44

1

16

4

32

137

12

42

1

1

1

15

2

2

2

2

69

1

1

2

28

25

12

10
1942101

491878
15

13

177

2

9779

221

6552

211

128

8149

116

124

1

17

71

318

19

2

2

1

1

1

1

31

15

2

3132

13

11

1

551

223

15

34

144213

1

28

144141

4031

3

15

1492

2

4

37

1

1

31465045
20864

1

1953

20

15

15

1

14

1521763

6

2721424

285
3474637

990

36

684

17202

17202

17202

4559

484

13

462

996
11

528

10

248

2

2

17

17

3218

3218

63387
2

439

62946
1

6508

138

2299

2299

11

1

1

391

1

1
291

112

178

1

1

1

1

12294741006265309659
577091104

2104
1838639044731

3918

3918

3918

121172033

2102001

2102001

2102001

2102001

2102001

102

102

102

102

109730

109730
4

1
4192

17

15

15

15

2

2

2

3112

24

24

24

15

15

15

1

1

1

1
12

2

2

1101

121

121

121

121

8

8

8

8

21025

21025

33
1620

114

114

1

1

1
2

1

21
11

1

41

41

41

14

14

14

3542

3382

222

2

2

22

22

33

11

11

22

22

13

13

13

16

5
16

10

10

1

52496
1836936862686

24160195

24160195

21157180

259

259

259

53
19152171

11326

11326

9434
32

21

1

28

11

512

4115101

4115101

5157

5157

3315

2
1212

2

2

1

1

17

2

2

213

213

213

8026091688
310321

331193620

331193620
2583

38037

38037

38037

62094
2

51142

51142

1932

42001

42001

42001

216126

216126

920715

920715

920715

114339

114339

114339

6135495

6135495

6135495

26807582

26807582
1111

1161
51566

4632

4632

773

773

4120526

4120526

4120526

714820
1

36111

36111

4869

4869

61679
222

1857

3602

3602

21086

21086
21085

1

19714

19714

19714

18506465

18506465
12

27433

27433

27433

8
1226735

719118

719118

56817

56817

216376

47
216376

24201

8168

213721

213721

213721

139616014
1224

33366
21

11

11

11

11

121

121

121

121

26223

26223

69

69

43

43

26

26

13916224

13916224

12216172

12216172

12216172

170052

170052

170052

41391970

41391970

41391956
22

1
152

21

21

31

31

766

435

131

2

227

227

227

4127819
4

417

662

662

4113110

4113110

14

14

14

14

115709587
12

2210024

2210024

2210024
1

197921

197921

3203

3203

46249457

32
34152409

476

476

476

71541

71541

71541

13
993133

42940

42940

21966

21966

33227

33227

221158

221158

221158

4765

4765

4765

421

421

421

173

173

173

129748

1
129748

8279

8279

47038

47038

4
47359104

712842

77017

5282

5282

24215

24215

5825

5825

5825

210

210

210

210

2121419

18
1719717

5927

5927

119710

119710

4172

4172

4172

191133

191133

191133

191133

10169

10169

10169
1

7109

51

51

41

41

122

122

136

136

35

35

35

328723

328723

13
328723

1
1098

1

513

411

41

41

122

122

217515
6

41

41

410

410

3125

4

4

3101

2

2

1156

351

351

312

312

324

324

1
324

31

22

22

85251732910188
12110311001652303824

37871122231457804
263314861053

2664974497
251

3831426

3831426

3831426

3831426

3831426

3831426

59208044
25

42163937

42163937

42163937

42163937

42163937

42163937

42163937

174167

174167

174167

174167

174167

6633264
23

716927

716927

716927

5914037

5914037

5914037

3711832
5914037

15152

321

2

12

22

11

3756264

3756264

3756264

3756264

3756264

3756264

661661298

9
661661298

17387109

17387109

17387109
1119787

1

1

1

618921

618921

491265189

491265189

491265189
21284

21565138

2657247

2657247

8127186172
3444121121611983

2172733
2711446555702

237241383615
1152917

361810
87812251923

123545153
4529749

119916

4206

94936

94936

102

102

102

399

21

21

5263

5263

72638

183

271413

271413

271413

171645

171645

171645

51869

51869

51869

25751

25751

25751

5996171582
423430

426799
125213202

26154
474723

1106

121

121

192012
541

842

6129

91613

91613

101417
51315

3

11

2

1

72817

72817

22218
18

7

11

21

106

23

81915

81915

203102650
9621177

81720
3345296

1917

1

44

11

11

4414

4414

2

3210

215

115

7645

6512

1170

1170

34

34

239
2311

1

1

14954

14954

24325

24325

2615
312183

12

134

137

41

41

10

216

13

5614

22

1113

51334

457853
26167

103345

9291

2330119

134251

134251

161517

161517

29253
42889

334

36

7326

281821

281821

3548183

3548183

2216133

2216133

6051824608
15371

394960464
361620

543122

543122

543122

338413

337138

337138

342651

342651

11750676
147616290

5257

185079

735128

5711630

798043

798043

798043

117747100

117747100

117747100

571623

571623

571623

571623

6311763

6311763

6311763
221315

184015

101912

101912

61412

7319

7319

283713

283713

283713

283713

13726198

13726198
30817

12520

12520

12520

859

859

1028

1028

38691
617104

1

1

97

1

1

51

71

1

2

16120

16120

13478597
1

8963968

8963968

8963968
211227

3331720

3520041

4514628

4514628

4514628

301
35579673

24329

24329

24329

30175644
55989

13261

231116
21188

11

127

47364
6533104

142936
7283

15

7

14

3

26

111

2

1

24

10147
10146

1

2931

2931

2931

4141

4141

39460

39460

10259

10259

28616
26412

211

13

251620

251620

251620

245254902054
1792625

18125175823
3437

32225124
27

29713112
30123

151538
70538

3

5

62

11

9937
116751

51

1

7

266

217

2249
23512

113

17769147692
11221679

8528

8528

303122

303122

470328
316

4481
1

448

1

1

1726

1726

11

12

12

11

11

4394115
104416

19004

14505

47633
43333

11

21

1

1

1755

1755

1214

1214

7336
761767

39211
84216

6

312

83

11012
524140

20330

2118

8015

8015
6615

14

2312

2312
512

10

1

7

1833

88315
65211

1

2

1

1

6

2

1

111

12

21

4

1

22334

22334

1174230
1334332

39

10

16

21

461

282

3013

3013

3013

171332

2358

2358

182718
5220315

182117

15721

309246

309246

309246

529327

529327

137979
3

42241

11241
42241

3

15

12

1

92738
18230

6855
3444

2

3211

63

63

12047

12047

46516
24215

21

21

2

1821

42126

42126

908

908

9371015
18815631

1114

11

7

1

14

880

9445

4

1

112

31

15

6316121

481
6316121

25564

25564

17126

17230

17230

611
171245324

163
164729274

301135

301135

18316

18316

86159

86159

71238
428443

176

114

1791

11

265218
1147518

263

1

8

6103

32721

32721

32721

27828

21619
27828

113

516

5132
43838196

241523

241523

241523

21820155
82423

17426

17426

15

22674
5327

10234

7113

7113

36513

36513

36513

2513

2513

361

361

14516

14516

14516

1193

1193

1193

1193

2221
3997181562

65
463388

21311

21311

23848
43672

11124

87

3512176473
1932049

146196
114196

15

8

9

41413
39213

22

4568
63819

11
1128

6

517

73

6

13

13

314250
667273

1912

16221

1768946

1768946

6727

6727

6727

2341
1727

11

1

49

4

17216

17216

25116
149

53

623

63012
73018

11

5

20532
1346

2123

2

51

247826
106515

1

1

82

1219

1012

221832
201523

6

233

1223
2253

1

2

4

42

1202330

1131415
1202330

2

24

1

3514

1822

1822

24141

24141

1

1

21713

21713

32223159

32223159

32223159
5

1451489

1451489

1451489

172970

172970

172970

8035702116
967

81743044

81743044

81743044
3221

4977722

4977722

4977722

28835121

28835121

143810928

143810928

19747
143810928

743789

743789

4982712

4982712

568415644

568415644
191

463614021

463614021

463614021

10291523

10291523

10291523

28916456115748
35010815

836822555
24301

334152273
5

6238
1391383

29528

29528

48547
28333

1218

816

28807

28807

28807

16754183
823477

428

428

17136

17136

12

12

11111

11111

11

11

241119

241119

1439

1439

13220

13220

526544

332813

332813

332813

193731

193731

193731

348523236
189

502216

502216

502216

502216

7030518
2

481729

481729

481729

221319

221319

221319

354033

354033

354033

2511925

2511925

2511925

47422

47422

47422

10324122
792075

6220

6220

42

42

42

1

1

13225

13225

78521

78521

78521

78521

271378

271378

271378

271378

271378

84879551
28777845704865

82393
125211721857

19274
410444133

794820

794820

421822

421822

827037

827037

769510

769510

383217

383217

7415423

7415423

1471817

1471817

1471817

1072619
1162619

4

5

4712020

4712020

4712020

3172
22511972

25397

25397

292611
805828

21129

30208

891535

891535

453036

453036

453036

5326424

5326424

5326424

1271121533
99

441830

441830

41711483

41711483

331420

331420

15932028

15932028

15932028

15932028

216201137290
4711123

1164394

1164394

1164394

1365245130

1365245130

1365245130

677433074

677433074

677433074

3691847

3691847

3691847

56902342
112

47981621

47981621

780721

780721

10986342

10986342

10986342

10986342

10188544

10188544

1

1

1

10188444

10188444

10188444

7011935
21

414818

414818

414818

414818

277017

277017

277017

418625251
4

408716223
3

458471
39979153

73211

75936

67228

264017

87770

95928

95928

95928

718938

718938

718938

718938

399286074
2224

2
398465029

9894920

9894920

3885519

3885519

60841

60841

60841

6029
65603321837

404
851071

27425

18246

62182751654
5208395

93759

93759

97121
97120

1

1037120

1037120

1358180

1358180

8

8

3919508

71386

71386

14919126
73936

63833

13257

13216163
17817179

204

26112

132649

132649

18420282
40439449

45421

45421

77550

14122

615

615

57323

4110

62754

712

25130
4110

51

1619

168754
167754

1

112

112

12214102

12214102

12214102

1081326

19745103
413

663087

663087

901216

901216

16831
6772373290

48813104

48813104

48813104

100422426

100422426

100422426

52169103
5861

1391719

1391719

1391719

129845

129845

100130

100130

95378
85377

8

21

411827
45916456

30063917

30063917

11741712

11741712

388210028

388210028

388210028

388210028

1
17398235

225
832085

38510

38510

38510

431370

431370

431370

8978150
111

6538118
556

351787

251625

251625

233931

233931

233931

116503986

116503986

61
116503986

105366

105366

115393619

115393619

115393619

1913249

1913249

1913249

1913249

1913249

1913249

1913249

787606724187

787606724187
35189791

28198340
1

1021122

1021122

1021122

12140166

12140166

12140166

63652

63652

63652

551320518171
2247166

424227816705
1368594

33261379
32783

124531

12414

59943

21217

22369

831122

142141

142141

142141

604078269

604078269

604078269

53584

53584

53584

314097

314097

314097

2231111

2231111

2231111

102198

102198

163170

163170

13126308

13126308

13126308

41518
118349

23219

53612

53612

5377

5377

5377

9108203

9108203

9108203

2549480
18210436325

1312090

21194104

151211011

241141019

1822336

17981540

74130

2095962

898132
893132

2

3

63870

327487

52664

1058801300
710

152172

152172

147697

147697

147697

21239167

21239167

1536163

1536163

1536163

411289

411289

20318303

57191

57191

15247212

15247212

337126

337126

1334273

1334273

1334273

39921487

39921487

39921487

1440486
39921487

1162

2327

3649

3649

349

5175

2595

2595

1375

45116

118136

2463

4152

21102

401207235

401207235

401207235
25432

1956887

1956887

19585116

19585116

9411763163
104993

30209441

2057299

2057299

10152142

62862

62862

412480

549182629
43348

673115

673115

673115

143631854
613

45

45

111561636

3197200

3197200

1461134

1461134

1461134

1369
16388478

57994

3107139

3107139

3122130

56746

4816327494

4816327494
3453

2172762211

6806
2172762211

3579239

3579239

3579239

37
4365363

2228718

2228718

1835945

1835945

2639215

2639215

2639215

10784588
1814716

3313512

3313512

1016711

1016711

1613918

2016220
3025731

1311

3286

2181

4183

2613520280
93

3557734

3557734

3557734

3557734

2262934243

2262934243
269714

3235619

3235619

2326026

2326026

2866747

2866747

2856232

2856232

1740131

1740131

2440024

2440024

4819150

4819150

86832126
2938119176414488

362021990

362021990

66
362021990

301439931

301439931

301439931

657653

657653

657653

2549217826212785
464122691689

14710083407
218411

813175181
13836

41188
422363108

2685441

2685441

12139159

12139159

12139159

3877437

3877437

3877437

29289363

29289363

29289363

29289363

353831152

353831152

353831152

353831152

945038

945038

945038

945038

945038

2407585793
27

251
2112501584

28923115

28923115

28923115

861107
1811573468

1514082
1210974

11

11

1

2276

2

2220744

2220744

3942238

3942238

5639094

5639094

41353103

41353103

41353103

295057209
11832

11296691

11296691

11296691

17207386

17207386

2092869

2092869

2092869

2092869

2092869

25555309
729150182444

141109110

141109110

141109110

141109110

51089

51089

51089

35182090
1263

1981836

1981836

1981836

1597651

1597651

1597651

403875

403875

403875

403875

34115999

34115999

34115999

34115999

822998620
776118

391503271

391503271
842169

734957

734957

20373115

436030

436030

12684110

12684110

12684110

24735121

24735121
612138

828036

828036

1033447

1033447

502897113

613927
502897113

16106323

16106323

1482128

1482128

1487435

1487435

29221114
3912555695

4568

4568

4568

15712897
852

383741

383741

1118654

1118654

3418095

3418095

3418095

3451976

3451976

3451976

79186

79186

79186

3833859

3833859

3833859

1
4037558

2435255

16232

48647102
126

2432762

2432762

2430834

2430834

28635100

28635100

28635100

28635100

872396

872396

872396

872396

1751948

1751948

1751948

1751948

7110088453

7110088453

7110088453
441540

13231765

11119795

16194496

16194496

13298348

141232109

14616850693

14616850693
6771145

17167261

17167261

17167261

849909306
750752

17225253

21226148

21226148

24234162

15254891

15254891

41426
18175069

877235

877235

683628

683628

212748112
7201128

639311

639311

834473

330264131545

529967
330264131545

309240501335
713434268

232141101

232141101

30205075

30205075

22223778

22223778

996396

996396

112219

9102240

9102240

9102240

9102240

9102240

16179867

16176767
16179867

31

15138357

15138357

141261141

141261141

1038

1038

16163745

16163745

7

7

221622100

221622100

221622100

21152773

21152773

16107246

241671121

241671121

162064143

162064143

162064143

829472723052
37944168

1360281

1360281

1360281

1360281

234326641252
51

925899

925899

925899

385258275
225324011152

13136736

9

34735114

34735114

34735114
2149296

51709
1324318

8517

222

4632043

15193473

15193473

111329

111329

19208036

19208036

7223129
722343

86

21204257
7120738

1
32

12

1

44

44

7794
2

11

1

1

2

7623

2

3

2

3

32633
77499

2192

3171

1973

141

12

1081

13209635

13209635

27738

17211372

17211372

17179773

17179773

34646
16392463

1833
231510

844

1792

691

8181722
10275034

15569

13773

1982

1982

29711

29711

4

4

119414

119414

6124939
9180764

126516

22939

3442071840

63124174
3442071840

61278
61251

27

205047

205047

206631

206631

249629

249629

32327
42630

2

131

2114725

2114725

1722822

1722822

724113

724113

145437

145437

2913739

2913739

1513229
2116833

1112

5252

2437644

2437644

154640
155242

1

52

184444
275155

222

2

25

1

111

2

1

32

2025616

2025616

1220425

1220425

11075
1128514508

393434181
1257

10132379

10132379

16112652

16112652

1386043

1386043

724973322
931140

2125883

2125883

3148457
2129431

38913

710113

881343

881343

2083150

2083150

2127649

2127649

732067133

732067133
4114

2571539

2771146

2771146

2160034

2160034

1641070

1641070

1641070

1641070

485568217

428637
485568217

21147341

21147341

21147341

12182582

12182582

12182582

11198457

11198457

12015159516

12015159516
837616

960591

960591

960591

17212490

17212490

17212490

7299473

7299473

7299473

23212154

23212154

23212154

11203
32524593

11179041

11179041

20333549
20333545

4

24169499

24169499

24169499

281236131

281236131

281236131

281236131

281236131

17220260

17220260

17220260

17220260

221351312368

221351312368
1944

220771285213
72310215

25510717

25510717

17846
14519633

185512

185512

1105715
915315

1

184

9112128

9112128

4612136

4612136

14817211
88120017

530144

157

18872

1111926449
746113820

3117020
125

141567

158913

10251

1334

610133
3112478

504151

13111

152043

1269

2215

234748
60504

31111

143133

858310010

858310010

3923151

3923151
181056

181288

317

19149165

19149165

19149165

19149165

19149165

11583109

11583109

1035928

1035928

1035928

122481

122481

122481

254255136

254255136

254255136

254255136

254255136

28874283

28874283

28874283

28874283

28874283

28874283

37051842206
53306

31251410151

28012917
31251410151

28318321

28318321

28318321

1316
99845431

18610010

79933821

79933821

104925630

104925630

104925630

48635018

48635018

48635018

293834

293834

293834

132
52740249

1611899

1611899

1611899

1611899

35321140
433

822718

822718

822718

26718119

26718119

26718119

34793398

34793398

34793398

34793398

34793398

34793398

7323485053

34511
7323485053

3272134596

3272134596
1736

6319201
2892074532

1203

1203

2041
22141

2

1

11325

11325

6448

6448

110
119

9

5238

5238

1249

1249

3528184
20655

71436
4111

3218

3218

3218

117

11
117

106

337
3239

2

2

5654

5654

5654

13253
19871

12

3281

132

170

2123

7257

7257

615162
67142

22

218

3

1

129

129

2348
12

1346

16345
11342

53

1133

1133

48317

410193
8561121

618

1338
11138

8

15515

26239

18118

110

110

32644

32644

12111

12111

6338
77463

14425

529335
516125

345
348

3

3

10140

18

1

417

417

10240
11241

1

1

17139

17139

2381
114

1252

25

152
21358

4210

4210

4210

2146

246
2146

1

23596225

23596225
5181

1344
1673286

521732

521732

64826

64826

38324

24229
6346138

6410

649
6410

1

1

16681
723

3527

3527

6131

6131

171438

171438

222
13634221

30125

30125

23111
30125

54

210

10431194

10431194
291240

20476

20476

31719

31719

744

744

348

348

1447

1447

29130800461
3270

2283272

2283272

2283272

2283272

20819013323

20819013323

20819013323
1695019

34520533

34520533

34520533

183
526301158

25330688

25330688

26291270

26291270

21161
69349968

27160642

27160642

40177725

40177725

37305845

37305845

37305845

581068566

581068566

581068566

581068566

581068566

581068566

97309520

97309520
21

2859219

2859219
4128

14263

14263

14263

1021208

1021208

1021208

14
69248300

428759

352233
373041

188

1

3194

3194

3194

178

178

178

2216

2216

2216

16
26157241

71
2095211

1278114

81096

81096

55630

55630

55630

154546

154546

154546

154546

6392
154546

7416

7416

2228

2228

30231121

517411

517411

517411

2557110

185147

185147

7663

7663

138286432

211
138286432

3341114

3341114
12

12167

12167

12167

2113107

2113107

2113107

85139290
6

2
5225204

3210186
13

16462

16462

153124

153124

201318
53

846

846

7612

7612

3310886

185073
11935

51233

51233

1

1

2294

2294

155813
5325

442

442

1

5116

5116

5

5

15

15

189528

15
189528

63919

63919

63919

12419

12419

12419

21144
6763921003924606

154338

154338

154338

154338

676242833024493
313033

8014986

8014986

8014986
17163

306670

306670

306670

306670

336713

336713

336713

336713

93196538
675845652723434

67217511241460

67217511241460
123671560

212411
88750216472

3671628142

3671628142

3671628142

26395134

26395134

26395134

480222946

480222946

480222946

944
91466151

5993248
2112341

4735

3156

262

3063099

3063099

2473888

2473888

2473888

21404246332

21404246332
35891418

57901413

57901413

273013086

273013086

20984125

20984125

19361537

19361537

128514109

128514109

39761844

39761844

1197273

1197273

1197273

1197273

75526472159

48002822
75526472159

4100020759

4100020759

2972623778

2972623778

474009168164

1586149
474009168164

130232230
405872650

177165

453034

5318111

3415641928

3415641928

107506827

107506827

542322123

542322123

110153027

110153027

132793317070

394140
132793317070

5533550

265114
5533550

6581

3229

3229

5841

5841

7363

7363

5245

5245

2436

157

157

1384

1384

115480615226
88651072

5915278

5915278

5915278

1138401

1138401

1138401

4011173

4011173

4011173

1412341

1412341

9288407
34

247263

247263

6878140

6878140

2628348

2628348

2628348

1611870

1611870

1771973143
2311140

2139574

2139574

6969603

6969603

4214462

4214462

1226732

1226732

1038632

1038632

2014464

2014464

2014464

3792030

3792030

1419415

1419415

1419415

4287139

4287139

4287139

1729272

1729272

1729272

36371904
39

2012829

2012829

16251036

16251036

2612330

2612330

2612330

5647187

5647187

31512994

31512994
17711904

1320

28

82161

411

1811391

1811391

2252585

2252585

2252585

2812482

2812482

79901154
2

3734777

3734777

3734777

4256375

4256375

4256375

64311062771

64311062771
6548

1242
61109680

1451158

1451158

1451158

3127284

3127284

3127284

1619196

1619196

1619196

2247186
1

1012134

1012134

1012134

123551

123551

123551

1447281

1447281

1447281

1447281

362034
5408981576

5438117

5438117

5438117

5537371

5537371

5537371

1

1

1

81320113

81320113

81320113

7326589

7326589

7326589

7866446

7866446

7866446

6942161

6942161

6942161

94109245

94109245

94109245

64814901484

947
64814901484

3
118232453

6129245

6129245

6129245

57203205

57203205

57203205

371293748
121146

11154136

11154136

6299148

6299148

6299148

9346223

9346223

9346223

9383195

9383195

9383195

7511
150961276

53743125

53743125

53743125

90213140

90213140

90213140

1211778111

1211778111
221

4549630

4549630
3739624

5482

3524

74128080

3152831
74128080

2138024

2237225

2861624940

2861624940

2861624940

2861624940
811219

133651754
31610

80391696
52

1
22179533

8129476

144957

23579

23579

2710766

2710766

84386

84386

5024448

136421

136421

3718027

3718027

9164476
7393

4626829

4626829

3833744

3833744

3610738

3610738

3610738

1811053

1811053

1811053

807220

807220

807220

807220

807220

807220

807220

807220

34158051

34158051

34158051

34158051

34158051

34158051

34158051

2269125

2269125

2269125

2269125
151

10864

10864

10864

115660

115660

115660

111710706767204111
1173192554085

66704153

66704153

66704153

66704153
23439

928335

928335

2816533

2816533

619163
621376

8

1413

304016521723614
2603132629

461083207

461083207
51

26836110

26836110

26836110

2024296

2024296

2024296

2513950608
713

100420295

3013789
100420295

2811189
48185135

61214
166

51

58

1
1227

71

156

9

7

2138

522

51413

3

3

112

144853

144853

85018
63414

13

1134

1443517313
4873

40609132

40609132

40609132

49133395

49133395

49133395

49133395

51148883

51148883

311877100

311877100

311877100

311877100

311877100

27412624667

743859
27412624667

452832122

452832122

48230177

48230177

48230177

151
614765195

37258785

232127110

47112087

47112087

47112087

661168127

661168127

661168127

18679121

18679121

18679121

18679121

18679121

981692633

5060333
6921

41721

577

577

1319118

16320

16320

61276

61276

13582

13582

331132

331132

32159686

32159686

32159686

191113875019298
1344518609

738719339858
1065277295

331479108

331479108

948799637
451639

181553239

181553239

22179187

22179187

201725107

201725107

765929
303214165

16120168

7135468

425195184
277374466221

645832

181666113

14309494

14309494

253115109

5010263761
12177277

41323218

111657111

111657111

3104076

3104076

132537110
6123454

3

137012

690642

242

433049

433049

31604120
148715

153546

158259

126974

192210115

192210115

13283059

2022993468
7059334609

6415229
20864912

7582

4067

87785

5132146

128162

4246

3549

612

1434

282696144
13736

11110979

16121459

27485
15043

11213

49

311

59

19241371

1636249

39
1636249

422100

422100

1214110

1214110

12890791812
1228924

16541277
5537581329

13221897

11514411

15485544

615032459
302629184

747644

444763

948370

310525

114521

774752

340618
849817536

1574394

1574394

9280182

9280182

1246783

1246783

222432107

222432107

232968152

232968152

18410961901

18410961901
2133145

721397207
3057395

1535957

1535957

2746555

332938213

332938213

332938213

232811214

232811214

232811214

353484222

353484222

353484222

928153
21193361225

1716209
5515

8584

8584

46110
4475

2

19

124

593822235

716910
593822235

331614125

192039100

2122995

2122995

24209124

24209124

24209124

282094115
814779509

281566179

15920
16743

823

1647593

1647593

785
750544

31948

15721

317610

3
17235

245

14530

544383385992
431571483

411396412

411396412

411396412

698630907

131532197
698630907

192538292

172138218

202422200

22928
602853843

341632394

241192421

241192421

483050256

483050256

483050256

23680346

23680346

23680346

271859248

251464200
271859248

26332

213216

55669243

55669243

55669243
3

55666243

13302119

13302119

13302119

11746701386
411156294

36897710

730081

730081

1034978

12912107

12912107

111056116

111056116

111056116

312477229
4812658749

89735144

89735144

9446376

1003664713
116862

1028891

1028891

2238389

2238389

291401152

291401152

291401152

281524319

281524319

281524319

82753488

11404
82753488

1328251

1328251

1328251

89621
58431433

1049103

1049103

48768

20104103

20104103

1695138

69677863

10596
69677863

13118129
23319174

59621

510524

510524

14310115

14310115

14310115

2243478
6283

718194

718194

923201

923201

3247416390132551
22981951275

212128082134
25833

4405106
192119331846

573089596
725565

14752176

14752176

241163166

12919189

12919189

13011
432137307

221038159

201069137

201069137

394862519
29714

231722242

143043263

143043263

231308201

231308201

231308201

26132117

2466
26132117

104653
186975

61510

55

22

213

2

61736

61736

18817255

18817255

18817255

18817255

135033899310073
1199316369

27207396

27207396

27207396

385244832804
1456156

889123795

238630
889123795

465827383

895759
465827383

12150288

81714109

181654127

221014142

221014142

141674206
181896240

477

1353

1

110114

2389

1

271146731461
1543748

2797
823773537

422348296
123

18677188

18677188

241659105

241659105

22626135

1672099

1672099

1078255
17410463876

1156643
534476376

111261180

111261180

312649153
695

9122481

22135667

45625135

29150484

29150484

373076226
23123082

434512

434512

71260113

324119

156
12126492

33079

33079

222102

222102

337127
18127

229

332178

332178

173
847783542

16624
425511325

9123682

131605103

131605103

202504116
1606

10111364

10123146

22800143

22800143

22800143

20145571

20145571

20145571

8711664564
13178

273216294

273216294

273216294

383136107

383136107

383136107

214995155

214995155

214995155

136213601252

20216377
136213601252

274703265

112123108
274703265

447524

693240

257352

460041

323519402

323519402

214442167

152008117

152008117

214525224

214525224

2652424092144
2410243144

8974642746

8974642746
1311420155

7753184

6264140

16978590

912057101

1613696105

1914962105

3255066

1717260266
5773

10453295

10453295

712151168

712151168

23564297

23564297
23557096

691

3

112134622891
1618925142

2425638132

2425638132

1315049118

914890105

1024954123

1024954123

2316278123

2316278123

1718888148

221774188

221774188

221774188

221774188

111
303139172

1681479

1681479

1681479

1681479

13231493

13231493

13231493

262
10313684609

20119787

20119787

8312461520
19174165

103067146

103067146

172793104

172793104

122286100

122286100

252574105

252574105

252574105

44811150

910111
44811150

1690

1690

7466

7466

2499

2499

59161

59161

59161

411128

411128

1837
212321

18

13276

62158

62158

84116

84116

481227183
32414

1462294

1462294

1462294

2156065
3158175

311

311

1

1

21

21

21

494
36

1

1

2

2

12

12

11

13
131

1

1

31

31

22

22

2

4950101245

4950101245
761157

282216125
116

14311396

13184723

142283963
103

31555060

31555060

11728800

4641197011045
4197

951160458
136

43498188

43498188

43498188

36639152

36639152

36639152

1520112

1520112

1520112

17331448
3651079110580

16350351

16350351

512118695
21825

23664358

23664358

261436312

261436312

8939431384
2114

26789480

26789480

251331444
633133890

241047299

22946

1021236

431465

6484320

6484320

6484320

22780413

22780413

22780413

2115767

2115767

25374313

25374313
24373312

1

11

16213
152604639

61101571

61101571

91342855

91342855

19406702
4181

11205232

11205232

8160389

8160389

46538513
19126153

99087

99087

49
18322273

10181

18217183

11
381050735

11280216

27769518

27769518

3012159

3012159

3012159

3012159

3012159

552282842802
622220

389223132090
753950

653666464
59211

342056199

342056199

261518254

261518254

261518254

342709231

342709231

342709231

849838
15210609773

483807221

483807221

966304514
2384572

272833190

462626252

36812117

36812117

36812117

866100

866100

866100

7
873912355

261374173
261377174

31

612528181

612528181

1575749692
513624

3610481

187257
3610481

171315

1199

2948580

2179
2948580

2030539

716332

23130992

23130992

23130992

2341865

2341865

2341865

2530563

2530563

2530563

162992287
152199214

136017

136017

43356

43356

21
1362621741

67118915

67118915
26

717291

717291
612181

1565

45

192782
6099618

106175
510

2683

373
382

9

57188
1

34109

2359
2378

17

2

2659173
133092

21

21

134

134

1

1

4317

4317

22

22

11

11

33

33

3
2

1

214

214

11

2

2

521

521

5711

5711

12

1

11

91676

91676

91676

91676

14014
60126749

1326332

1326332
134

1438

1438

77136

77136

448

448

41176
41033

133

10

3213292

158109
3213292

4371

4371

1

3175

6126

6126

32

18

12

6

33567
1627

3

3

2522

11415

11415

8517
111244

373

373

24

24

131122011281

131122011281
330653

606204587
852963

171471157

171464157
171471157

7

101701136

101701136

252503231

181072411

181072411

181072411

3248675

3248675

3248675

184133155

184133155

184133155

2020876

2020876

2020876

2020876

2020876

743298820
7

262858269

262858269

262858269

31361149

97583
31361149

1313946

914720

1772402

1772402

1772402

1772402

1002125737335763
34817752390

841309302

841309302
137

39283108

39283108

39283108

2380694

2380694

2380694

2121793

2121793

2121793

20512522733

728100
20512522733

221
876179

464650
182437

19

146

987

391328

391328

1324561

1324561
1216170

15

5320

243

117

2

4

3119
170560830

1527631

1527631

11544

11544

101053

101053

8425353
12638693

37

4220

2129

33143

525

6237

113

13461

515

7817

7817

7817

101372

101372
7825

119

2438
35

13

2

228

2715673

2715673

2715673

111377

111377

111377

544191
54

19528
15314

14

14

211

211

29

29

163027

163027

14632

14632

659233

49649
659233

11228

51156

1423681524

1423681524
223

1053221140
15953

4852

624109

624109

62558

52747
103795

1520

1212

3316

3421
2465439

629134
41964

24

24

17

134

134

1545

1122202

1122202

2559

2523

2523

1551105
123486

31

31317

11

1

1

844161

844158
844161

1

2

175968

175968

14
1120280

55120

55120

614156
2657

2222

2677

262481

262481

262481

87368616

87368616
2346

1044183

1044183

1044183

1993143

1993143

1993143

137388

137388

137388

43155156

43155156

43155156

42530755315
108133

29
12621921184

21027
351577505

715108
121537261

192

6

4151945

34

6

7883

1422134
8848

3235

55

3730

16

68576331
31636

1410435
2623997

35829

97733

18151150

2117048

2117048

510174

510174

510174

1827165

101487

101487

81378

1780412

1780412
211

723248

51666

51666

34187

34187

427239

427239

427239

1644312

138
1644312

3572

1239202

1239202

47167

47167

47167

47167

5119288

5119288

5119288

5119288

1975252780

5367201
1975252780

61386

61386

1323144

1323144

41416

713483

713483

159658

159658

15
638

38

225

172388

172388

11438

17

21877

21877

191663

191663

111893

111893

51971

51971

51755

737297

737297

257451

257451

756914
536556

7202

113

2967

13

159

3

111

275059

275059

275059

275059

275059

38152415

1412284
5159

3894

3894

3894

63131

63131

5257

5257

5257

1913874

1913874

1566
32274371233

48513
1413783660

53
581747262

2569482

331048177

331048177

1123
25598180

1322765

1322765

11359112

2367188

2367188

2367188

31682117

31682117

31682117

4421874

4421874

4421874

4421874

1363380493
2725

61110199

61110199

61110199

25932157

25932157

25932157

36101697

36101697

36101697

14337
12259135

210935

210935

65313

65313

35450

35450

2761367591

2761367591
252

389545
2091001390

51533162
1219627

1916763

1916763

2017072

100171105

100171105

100171105

2020278

2020278

236280

236280

236280

42299119

42299119

384
15122141726

2012
641268578

3960889

3960889

3960889

20618108

20618108

20618108

2
522369

4159

4159

4159

121308

121308

17187
121308

851

670

24
4759610

3045411
2155

1310141

37136

37136

122779

122779

1514195

1514195

1514195

3
25630425

728168

728168

728168

130
18602254

11521150

11521150

78074

78074

15219109

15219109

15219109

15219109

22
871531008

4636472

4636472
62097

229200
34122

17

17

1
3

2

19549
4312

11

113

13133

9

5463

7255

7255

6157

6157

39117534
48

629111

629111

629111

314151
1023186

3423

4512

4512

1817124

1817124

1817124

544105

544105

15
32184556

2594477

2594477
21031

848206
1764243

1818
818

1

512

3

3

3417

620203

620203

78974

78974

78974
47157

1

42

83

11

1611

767192756753
13

651081021
1221

51370

51370

51370

51370

57100
3640438

16898

41037

41037

224
1115203

2358

2358

712121
6986

11

118

10

1

1

13

1

1

31197
2353492

6832

914248

914248

51670

445

445

702191665729
1222531

28896133

28896133

28896133

662180455565
1001859613

9577186

74434
44887478

7205170

7205170

7205170

7205170

20327129

20327129

20327129

20327129

10311145

10311145

10311145

1794411677
49314

36630
251144293

9417133

13661130

301108140

301108140

1202066230
971304136

1257

142

2115

1

2101

6

182

136

1362566

2173

221

112

682495896

682495896

22709175
682495896

11410121

111929

11558295

413632

314444

16419200

11520118

11520118

18953112

18953112

18953112

12366291

1072837769
1441278

13814

2147495
761862506

22510

1439

921478

210923

1226945

613029

12331133

449

1122375

16525171

16525171

1631288

1631288

1631288

2028392

84943
7825451245

7375157

7375157

13590236
10388170

310028

10238

501531809
12627

16547202

13367205

12291103

8300272

117612
302109533913

19524115
24083583738

472176300
3471

17989217

17989217

27114082

27114082

542051267

542051267

542051267
2160374

129135

441361

1735745

1138752

201082010

870389
201082010

32180

31367

31367

2

12372

10034991046
341114144

23947587
6401128

10335326
10315129

7159

1338

7211133

7211133

940053

940053
736636

2188

169

17312

17312

17312

8237126
2

2441
321

1918

1918

22

22

821185

821185

1

1

2260379

2260379

312445

312445

165
612419163

39192087

39192087

2248371

2248371

2248371

9507384416629

9507384416629
26566815583

1315124
44180463442

1492214

1492214

39365361285
42716429424

5233

3

711
21

1

5

37828

3211

3211

39

5

25011

10

364

364

11273

42

42

919

13

13

3713

3713

71

184

2312

51

14145

51321

51321

83628

39
50112

33

819

12

3355

2

1

1

21

1772

218

2122

11345

11345

1331

47
13

27

7

37220

37220

1611

1611

3515

3515

23114

23917

61378334
37

157740
2110072

125

41615

1512

20145139

20145139

2149

1812967

1219276

1219276
8638

6226

4712

23138171

23138171

23138171

2417351
54301177

31057
11055

1

21

3177

3177

2410162

2410162

156529
66255198

4115

4115

4858

104433

104433

3312773

861
3312773

97350

164822

92367419

3962352
22

1733233

11041
1733233

7265

7265

5

5

135

135

92087

2029117

91550

91550

91550

21213

9254

5330567

1916441

3414126

136712
74493329

23129258

23129258

1314318
3829759

117317

72510

2336

5238

1421423272
2422557

1516234
9846

53618

14210

69778124
5153

2832867

3643554

1713426

1713426

1712431

1712431

9191102381
14196

778709168
19258

1820338

1820338

70440097
515914

187619

187619

28319738
14

349413

2489925

3526826

378125

378125

2512276

2510143

7715188
3710151

261813

143224

364
3073373

2699
551

91

1247

42745

42745

5164
1962

3

292

134411
152715

2

9
10

1

634

634

486927

486927

486927

1164
53354107

2926968

2926968

236935

236935

175545

175545

175545

175545

7296401
79

3363310
1240123

1227
12337

1

18

1

1

1

1

920150
81219

1152

116

12

561

171714
323382

3723

3723

6519
12945

1

5426

50208155
279775

57

11
2310673

1710366

536

4046174
217

221673

163084

163084

163084

11324071
1

688822

688822

4515149

4515149

75199

75199

24155

5144

5144

51259

51259

51259

21
343995

92155

92155

231839

231839

3715848

3715848

3715848

3237818

3438
3237818

9350
9361

10
11

1

41103

41103

4675

4675

32190

32190

11649

32202

32202

53100

53100

4423

4423

4423

101829

101829

1185
457283323

238219
156610

252

6117

5297
423193299

396128248
22794205

243

243

11

11

1

1

3

34

34

3

3

231
2653

1

1

1

1

3

3

12

1

7456
9756

8

15

931

1

1

211

211

1

18111

18111

528
938

21

2

13

13

231

231

223644

223644

21520619242
267718343

5116423
7320956

11224

112329

1658314454
18724214130

507527

377114

377114

1532710

24284

44851
8341931

1231

1811129

613

21

28

461313

461313

117135
78411158

1193
1203

1

3714744

3714744

109226

109226

62023

136127

3972478287945
51717

10895093130
9137

392153

392153

392153

392153

392153

273585

241558

241558

32027

32027

41334
10144402985

218
1402471716

461641527
75746

9320

9320

349
3522

113

49180

3878

11102

414

414

1682

1682

16611113
62629

6

1

11

25

31

20

1

3

1

12

21

1

12

11

27

27

4565

1

10422

1

15

2

21

12

5

3

14

1

22

3

11

117

41

5329

5329

2621

2621

4411
6317135

61181

61181

13553

13553

194822

194822

194822

101724

101724

101724

4311555

4311555

4311555

4311555

827651180
5218

6218562
4472

135162
246248

466

419

311

154157

19485

19485

98106
46

530

530

4470

4470

75137494
4256

4112

4112

1268

1268

31543

31543

2112
11297

435

435

5150

5150

4540
84670

117

793

20

25217

25217

7110
5701141

1

5623
14

141

3

200

65

2

140

1127

1127

2878473024798
9253139

403318

403318

403318

403318

403318

540436
490330371717

2371425
34919690602

1043614
1727294239

1977735
1234759165

31118939

31118939

16101731

16101731

57177660

57177660

39209960

39209960

39209960

45206380

45206380

45206380

45206380

1099619258

1099619258
37307569

472816

24247775
32163

253928

19172244

44333998

593572502

593572502

593572502

593572502

593572502

779371577

779371577

779371577

779371577
24186531

143163172

394343374

8282472

8282472

8282472

8282472

8282472

2
2771539615

125770376
117

4627358

4627358

4627358

1348117

1348117

1348117

4040459

4040459

4040459

2544135

2544135

2544135

152767239
292

286069

286069

286069

4638868

4638868

4638868

155179

155179

155179

6125921

6125921

6125921

189438

189438

189438

1
66125315

187116

187116

187116

187116

48118198

57
48118198

101255

101255

38101136
51319

173978

164939

771011100
2

5849961
31

1017924

1017924

1017924

2918717

2918717

2918717

1913019

1913019

1913019

1951039

1951039

1951039

1951039

5145339587
24592

3814561472

3814561472
3746726

2941834
3153337

1572

1581

3422227

3422227

6396085

5260094

4653427

4653427

5420847

5420847

3849163

3849163

2654666

2654666

109719113

109719113

3320251

3320251

7651762

7651762

122247691297
16438

111342201211
1041

7654710

7654710
7632191

381

19438

10274162500
6716923

9161257

9161257

221104185
8932532

4567

12866046

10041049

10041049

393722

393722

191067

191067

5918621

5918621

6752337

6752337

394417

394417

26970966
3

8257541

18413425

18413425

5642156

5642156

7738254

7738254

7738254

7738254

1612424

1612424

1612424

1612424

247123

15367

15367

15367

15367

15367

15367

93516

93516

93516

74162245

74162245

74162245

251
74162245

122331

122331

122331

1320100

1320100

1320100

407964

407964

407964

73549

73549

73549

60727220

60727220

60727220

11
60727220

1856102

1856102

1856102

2453740

2453740

1114
1813377

107552

107552

107552

74721

74721

74721

157810952009

354
157810952009

13428071043
212

1295774763
4413

251
9191269

401715

401715

2950244

2950244

20199

20199

323547

323547

114344118

5622755
337116

159531

8618

188223

188223

403540

403540

1054300316
163653

182920

182920

7531325

7531325

377023

377023

148728

148728

1

1

387435

387435

1
11

1

1

3163103

3163103

13628

13628

4532278
6

2217165
13124

27
21030

2

1

20

10

1211

1211

112

112

57
5151

114

30

140

140

15
17

2

718
239113

124

124

2416

2416

326

326

14129

14129

17039720

17039720

17039720
55595

2013144

2013144

258253
5216322

5217

57

21135

110

28394

28394

15265

15265

63244242

47
63244242

2124
3497212

94275

94275

111876

111876
91476

24

82150
122557

132
2

13

315

315

2514030

2514030

2514030

8617233

8617233

8617233

8617233

8617233

8617233

8617233

28425614

28425614

28425614
6

1549357
61

1244264

1244264
5481

2319

2319

214118

214118

2646

2646

24493

24493

24493

12416257
151

484142

484142

484142

21221

21221

21221

401093

401093

401093

127353

127353

127353

127353

127353

127353

127353

8619966

8619966

8619966

1032
8619966

25909

25909

25909

112933

112933

112933

302417

302417

302417

10535

10535

10535

32896109392147
722

47192274

47192274

47192274
51124

42622

42622

42622

42622

155297
1116

11927

11927

11927

132264

132264

132264

1829114

1829114

6740
1829114

2210

2210

29

29

22

22

454
457

1

1

1

324
122

2

2

1211

13

13

288

57417

57417

57417

57417

16078931022
1171

1386840790

225
1386840790

638
535008209

274166117

274166117

274166117

2677984

2677984

2677984

851810576
73821

22497140

22497140

22497140

36377145

36377145

36377145

9621168

9621168

9621168

11277102

11277102

11277102

211036231

211036231

211036231

211036231

211036231

211036231

42723
326892847829

724468

724468

724468

724468

724468

724468

692298654
1132

532138471

532138471

532138471

532138471

532138471

15147181

15147181

15147181

15147181

15147181

3260927884

3260927884

3260927884

3260927884

3260927884

3260927884
