# Supplementary material for: MetaWRAP—a flexible pipeline for genome-resolved metagenomic data analysis
Source: Microbiome. 2018 Sep 15;6:158. doi: 10.1186/s40168-018-0541-1 (PMC6138922; doi:10.1186/s40168-018-0541-1)
Supplement: Supplementary file 14 — Bin taxonomy. Distribution of the taxonomy among bacterial bins extracted from water, gut, and soil metagenomes using metaWRAP’s Bin_refinement module (-c 50 - x 10). Taxonomy estimated with metaWRAP’s Classify_bins module. (HTML 167 kb) [file 40168_2018_541_MOESM14_ESM.html]

Javascript must be enabled to view this page.

members
magnitude
magnitudeUnassigned

gut\_bin\_classifications
soil\_bin\_classifications
water\_bin\_classifications

147134235

1

1

1

1

1

608486
144134232

7

7

7

1

1

1

1

1

1

53466
2139

1827
1411

3

7

7

11

8

8
1

2

2

3

2

1615
2

5

149
113

2

16

1

1

7

7

115
15

1

1

1

1

7
58

2
4

1

1

1

46

46
32

3
5

1

1

1

1

6

3

1

1

1

810
171054

11

11

11

17

3
17

14
10

4
2

1

1

41

41

41
39

2

12

12

1

2

1

2
114

1

1

1

1

1

12

12

12

1315

1315
38

1

1

1

1

7

7

1

1

22

1

1

1

1

21
1

1

1

1

1

1
